# The explanatory power of silent comics: An assessment in the context of knowledge transfer and agricultural extension to rural communities in southwestern Madagascar

**DOI:** 10.1371/journal.pone.0217843

**Published:** 2019-06-06

**Authors:** Kathrin Stenchly, Tobias Feldt, David Weiss, Jessica N. Andriamparany, Andreas Buerkert

**Affiliations:** 1 Organic Plant Production and Agroecosystems Research in the Tropics and Subtropics (OPATS), Faculty of Organic Agricultural Sciences, University of Kassel, Witzenhausen, Germany; 2 Animal Husbandry in the Tropics and Subtropics (AHTS), Faculty of Organic Agricultural Sciences, University of Kassel, Witzenhausen, Germany; 3 Independent Researcher, Lauterbach, Germany, http://david-weiss.com; 4 Tropenzentrum–Centre for International Rural Development, Faculty of Organic Agricultural Sciences, University of Kassel, Witzenhausen, Germany; Universidade Federal de Pernambuco, BRAZIL

## Abstract

The distribution of silent comic illustrations can facilitate the communication and transfer of scientific recommendations about sustainable land management (SLM) to local communities in countries where many people are illiterate. However, since there are cross-cultural differences in “visual languages”, visualization styles need to be carefully selected as well as locals’ comprehension of the illustrated recommendation evaluated systematically. Three agricultural recommendations were chosen for comic-style illustrations, distributed to six communities in the Mahafaly region of southwestern Madagascar and evaluated using a three-step, interdependent approach. The silent comics illustrated (i) composting of manure and its application to improve soil fertility; (ii) cautious utilization of succulent silver thicket as supplementary forage; and (iii) sustainable harvesting practices of wild yam. Results revealed that general understandability strongly depended on the community that was surveyed and on the environmental subject that was illustrated. We found a strong relationship between the general understandability of comics and the divergence that exist in communities’ socio-economic structure. Education level was an important factor that explained a better understanding of respondents for the comic illustrating compost production, but not for comics that illustrated sustainable usage of silver thicket and wild yam harvest. Willingness to follow the recommended practice was impaired when respondents valued no change to the improved technique compared to the common one. Effects of respondents’ socio-economic characteristics on the implementation of the recommended practice could not be clarified within this study due to the small subset of data. Based on the evaluation of recurring comments made by respondents and interviewers, we conclude that comics can be a useful communication tool to increase locals’ awareness and comprehension for SLM practices. This, however, requires that drawing details used to facilitate farmers’ ability to adopt a point-of-view inside the comic story are used thoughtfully as they might interfere with the central message.

## Introduction

A key component in meeting the challenge of eliminating poverty and ensuring sustainable and equitable food security in Africa is human resource development through knowledge building and information sharing [1]. This means putting peoples’ knowledge and information, as well as the variability among stakeholders in terms of need, perception, awareness and demand at the center of agricultural and rural development efforts [2, 3]. It is critical that farmers know about sustainable land management (SLM) and intensification practices which are needed to promote multi-functional landscapes, ensuring biodiversity and related ecological functions that underpin crop production and other ecosystem services [4].

However, although today more people can read and write in absolute numbers than ever before, in 2016 globally at least 14% of adults remained illiterate [5] with highest illiteracy rates for African countries [6]. Hence, the poorest countries in the world, where basic education is most likely to be a key constraint to sustainable development, have very large illiterate populations [7]. In Madagascar, youth literacy rate (15–24 years) is around 65% [8] and thus comparable to many African countries. At an annual population growth rate of 2.7% [9], the demand for land and natural resources in Madagascar is increasing and accelerates environmental degradation [10]. The rural Mahafaly region in southwestern Madagascar is particularly disadvantaged due to its harsh climatic conditions, such as regular droughts and dry spells, low levels of infrastructure and high illiteracy rates [11, 12] leading to high poverty and food insecurity [13, 14]. With 97% of the local population working in agriculture, increases in cropping system productivity would be the basis for improving livelihoods [15]. Many of the environmental problems associated with rapid population growth and environmental degradation are not yet tangible for the local population but seen as part of the unpredictability of life. Changing a traditional land use practice requires an awareness and comprehension of feedback mechanisms that continuing everyday actions will have large negative effects on ecosystem services that outweigh the disadvantages of a change in behavior [16, 17].

Visual communication tools such as pictures, animated movies, posters and comics play a vital role in providing and distributing information on SLM in countries where many people are illiterate [18–24]. The visualization of consequences derived from destructive management practices and, at the same time, of opportunities that more sustainable management practices offer to peoples’ livelihood by means of comics can be thereby an effective method to overcome barriers in communication. It also can significantly improve comprehension and awareness of local communities for environmental protection [25, 26]. Particularly the distribution of pictorial comics without words, as it is the case within silent comics [27], has proofed to be a valuable tool for knowledge transfer in health and environmental education, safety instructions as well as of new agricultural technologies and innovations, particularly within rural poor and often low-literate communities in developing countries [28–31]. Silence in comics not only breaks down the barrier to environmental education for illiterate people, but can also enhance one’s awareness, understanding and the possibility of a conclusion of one’s way of life through the process of deciphering and deriving symbols, critical observation and the construction of a meaning of the illustrated facts [32–34].

Based on the scientific outcomes of a six-year (2011–2016) transdisciplinary research consortium of German and Malagasy universities (SuLaMa; www.sulama.de) that addressed environmental degradation and natural resource depletion in the Mahafaly region of southwestern Madagascar, key messages and recommendations for SLM were formulated by the scientific experts. Three recommendations, which were easy to implement for local communities, were chosen for comic-style illustrations, transformed to graphic narratives [35] and distributed within six communities. The central aim of using comic-style illustrations was to facilitate knowledge transfer from scientists to local people in order to increase environmental awareness and communicate scientific recommendations on SLM practices to communities in the Mahafaly region. Using a combined evaluation approach of qualitative data collected within focus group discussions and quantitative data of semi-structured, individual-based interviews that built upon each other, this study aimed, firstly, at analyzing the acceptance of local communities for comic illustrations as a communication tool for SLM practices by considering comic style and visualization performance. We secondly evaluated the role of education, socio-economic status and gender on individuals’ comprehension and awareness of comic illustrated management recommendations. By considering the structural diversity and divergence of local communities, we thirdly evaluated respondents’ comprehension and awareness on community level. To this end we assessed respondents’ perception on challenges and opportunities of recommended SLM practices as well as their willingness for adaptation and readiness to take action in implementation.

## Materials and methods

### Study area and local agricultural practices

**Fig 1:**
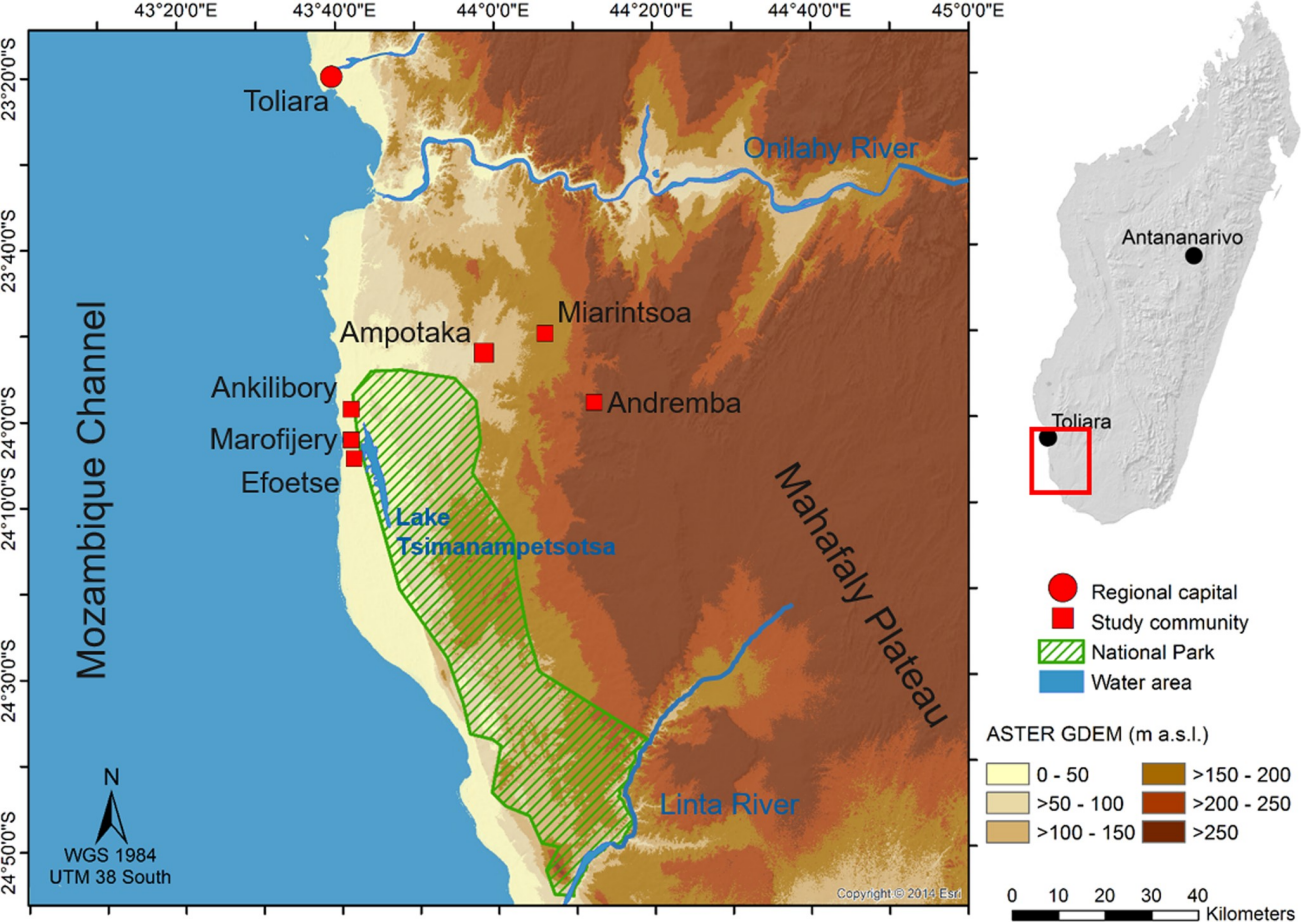
Map of the Mahafaly region in southwestern Madagascar. Original copyright source: Esri. "Topographic" [basemap]. "World Topographic Map". February 19, 2012. http://www.arcgis.com/home/item.html?id=30e5fe3149c34df1ba922e6f5bbf808f. (September 13, 2018). The area map was created using ArcGIS 10.4 (ESRI, Redlands, CA, USA).

Colored graphic narratives were distributed and evaluated within the communities of six local villages located in the Mahafaly region: Ampotaka (23°52′24′′S 43°58′38′′E), Andremba (23°58′18′′S 44°12′22′′E) and Miarintsoa (23°50′13′′S 44°06′20′′E), all located in the northern part of the Mahafaly Plateau, as well as Ankilibory (23°58′47′′S 43°41′38′′E), Efoetse (24°04′43′′S 43°41′58′′E) and Marofijery (24°02′36′′S 43°41′36′′E), located on the adjacent coastal plain (Fig 1). The Mahafaly region is characterized by arid to semi-arid climatic conditions with an annual mean temperature of 24°C and irregular rainfall between 350 and 500 mm per year [36]. The dry season usually lasts from April to November but may extend until mid- to late December. Livelihood strategies of the local population, which comprise mainly smallholder farmers and herders, are based on animal husbandry and the cultivation of staple crops such as cassava (*Manihot esculenta* Crantz), maize (*Zea mays* L.), sweet potato (*Ipomoea batatas* (L.) Lam.) and different types of beans.

**Fig 2:**
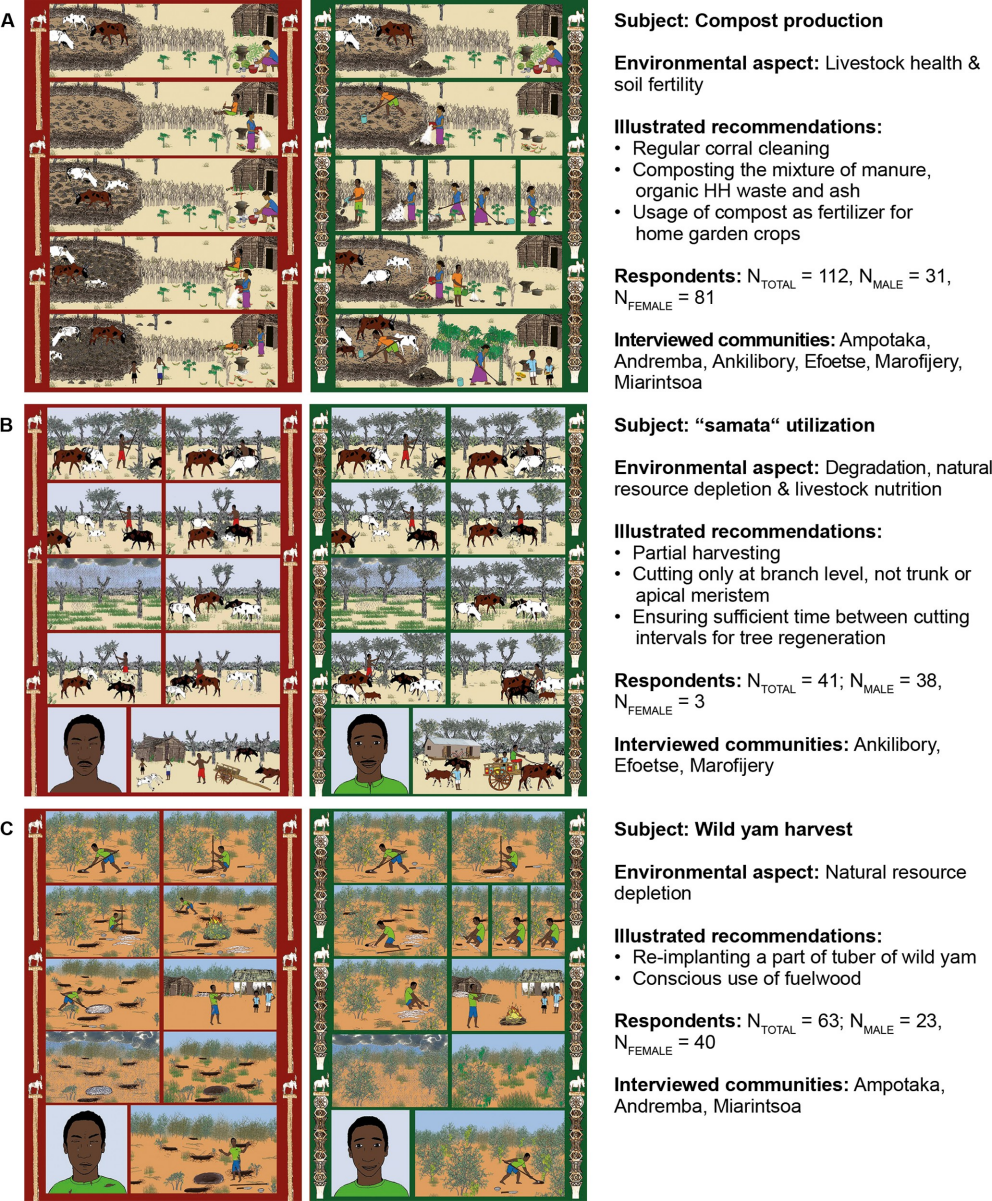
Silent comics communicating SLM practices that were distributed and its’ understandability evaluated within local communities. Left side (“red story”) conveys effects of unsustainable practices on the environment and local livelihoods, right side (“green story”) recommends sustainable practice.

Although agro-pastoral communities in southwestern Madagascar have high amounts of manure at their disposal from the keeping of zebu cattle and small ruminants, this organic fertilizer remains largely unused by local farmers for soil amelioration, a behavior which is partly religiously codified [37]. Given the low quality of the poorly stored (no roofing against exposure to sunshine and rain) animal manure, it is recommended that organic household (HH) waste and crop residues should be co-composted with the manure prior to application in local house-garden systems for vegetable production (Fig 2A). As a positive side effect, the processing of livestock manure after its removal from the herds’ night corrals may have beneficial effects on animal health. Corrals, where animals are penned together in high densities during noontime and at night, remain uncleaned for years and may increase the risk of epizootic diseases and parasite infections [12, 38].

The shortage of fodder, in particular for local zebu cattle, during the annual dry season poses a major challenge as this livestock species not only ensures the physical livelihood of the local population by providing draft power but also plays a significant role in both cultural and social issues [9]. In this context, drought-resistant plants such as silver thicket (*Euphorbia stenoclada* Baill., called “samata” in the local Malagasy dialect), a tree-like growing spurge, are of particular importance as dry season feed supply [39, 40]. However, the over-exploitation of natural stands of this species has become a growing problem. Goetter et al. [41] showed that improper cutting can damage or even kill silver thicket plants that meanwhile show a mortality rate of about 13–22%, particularly within and around villages. Hence, recommendations that were intended to be communicated through comics considered a cutting only at the branch level (Fig 2B) without damage to the trunk or apical meristems while ensuring sufficient time between harvest events for tree regeneration [42].

The collection of wild yam tubers contributes significantly to the diet of local people living in the Mahafaly region of southwestern Madagascar, but intensive wild collection threatens the remaining wild populations [43]. Altogether 42 yam species occur in Madagascar, of which 12 species of the Malagasy yams have been assessed as ‘threatened with extinction’ according to the IUCN criteria, largely as a consequence of over-exploitation and deforestation [44, 45]. Thus, conservation plans are urgently needed leading to sustainable harvest practices such as by re-planting a part of the tuber of wild yam and a conscious use of fuelwood (Fig 2C).

### Development and preparation of silent comics

To gain insights into local perception of art, different drawing styles and local symbolisms were studied in image-based interviews during a conceptual phase of graphic development in April 2015. Thereafter, color graphic narratives with highest acceptance and readability among the interview partners were prepared in accordance to local art styles. The graphic narratives produced for rural communities in southwestern Madagascar followed the silent comic style by renouncing on words to keep the stories simple and understandable for children and illiterate people. All illustrations were prepared to show the impact of different land use practices on the environment and local livelihoods using two contrasting scenarios that we call hereinafter “stories”. After verification of local understanding of colors [17, 28], the story that depicts a “bad” case scenario of unsustainable practices, often applied by local inhabitants, was framed in red (“red story”) and the “green story” shows a “good” case scenario based on a recommended alternative, SLM option (Fig 2A–2C). The three illustrated SLM recommendations comprised (i) composting of manure with organic HH waste and its application to improve soil fertility for cultivated crops (Fig 2A, S1 and S2 Figs); (ii) cautious utilization of “samata” as supplementary forage (Fig 2B, S3 and S4 Figs); as well as (iii) sustainable harvesting practice of wild yam (*Dioscorea* sp. L.) and conscious use of wood resources (Fig 2C, S5 and S6 Figs). The stories combined realism (plant, animals) and functionalism (symbolic meaning). The framing consisted of a traditional Malagasy element, the “aloalo”–a sepulcher art component (“bad shape” for “red story” and impressive “aloalo” for “green story”) in the form of a funeral pole sculpture, which ornamentation expresses the prosperity of a family or community [46]. Throughout the comic a coherent graphic style, naturalistic and with detailed background, was maintained. Contour lines were used as it constitutes an effective technique to render characters and objects more clearly and separated from each other [47]. Comics were published based on the Sunday page format [47] and printed on robust fire-proof, clay coated paper of A4 format size in a four-panel folded brochure, including a cover page with the “red story” (left) and the “green story” (right) as double-page spread inside. The forth page (back) presented key extension messages written in French and Malagasy language.

### Interviews

The evaluation of local peoples’ comprehension of the comics and awareness of environmental consequences was conducted in three steps. During a project workshop in September 2015 we randomly distributed the comics in abundant numbers to the local communities. During eight group discussions with six to ten active participants, up to 30 voluntary participants and two moderators, first impressions about the understandability, the general preparation and expressiveness of the comics were collected. The posed questions during the group discussions within the framework of the first survey are listed in S1 Text and S2 Text. The statements of the participants were generalized jointly and noted down. From each participant (active and voluntary) we noted the name and address details to contact him/her for further collaboration.

Three weeks later, the second survey that consisted of individual interviews was conducted in all six communities with a total of 216 interview partners who participated already in the first survey. Here, the number of interviews differed with regard to the topic of the comic but also with regard to the communities. The exact list of the number of conducted interviews for the individual comics and the communities can be found in Table A in S1 Text. From each respondent we noted the name, gender, age and education level as well as information, which showed us the degree of familiarity with this topic. We asked the respondents for their normal practice in dealing with the target topics, for innovations and whether the interviewee had already an earlier encounter with this topic through other activities of research projects or non-governmental organizations (NGOs). The education of a respondent was categorized from one to seven (1 = no formal education, 2 = visited primary school, 3 = finished primary school, 4 = visited secondary school, 5 = finished secondary school, 6 = high school, 7 = university). By asking the interviewees to describe the content and core message of the two stories, the comprehension of the comics was studied by grading the understandability from zero to three, as it can be found more precisely in S1 Text (0 = not described, 1 = partly described, 2 = described, 3 = completely described). In the same manner, respondents’ recognition of differences between unsustainable and recommended practice as well as their perception of consequences of both practices, hence their awareness was determined. We further asked how many different stories were seen by the interview partners and used this as a further parameter to evaluate individual comprehension of the comic subject. In addition, qualitative data were collected that comprised statements made by the respondents about challenges and opportunities of implementing recommended practices, personal observations of applied practices, their readiness to act in case they observed unsustainable practices by others and about knowledge exchange with their social environment.

The third survey, a follow up interview, was conducted two months after the second survey within the same communities and with the same respondents who participated in the second survey. We wanted to evaluate if respondents who proclaimed their willingness to adoption of the recommended practice during the second survey could successfully implement the sustainable practice in their daily life. The here collected data and posed questions at individual-based interviews during the third survey can be viewed in more detail in S1 Text. The evaluation of respondents’ willingness of adoption and the successful implementation of recommended practices was done using a subset of data that were gathered from the second and third survey. We here only used the data from respondents who did not follow the recommended practice at the time of the second survey (willingness of adoption) and who applied either the common unsustainable practice or recommended alternatives within the time between second and third survey (implementation).

To assess the acceptance and support of comics among the local population, we noted repeated comments from the interviewees that could not be covered by the questionnaire to draw conclusions about possible improvements in the production of information material about SLM in rural areas of Madagascar.

In the course of the multi-step interviews, all of them were carried out by a team of Malagasy researchers and assistants fluent in French as well as the local Mahafaly dialect and therefore able to communicate and understand specific peculiarities and terminologies. Interviewers were trained in a one-day workshop and briefed in detail on a standardized procedure during interrogations. In this context, the interviewers were instructed to inform the respondents only about the topics of the individual comics, but not which comic story the unsustainable (“red story”) or recommended practice (“green story”) shows.

### Ethics statement

The study design and sampling methodology was reviewed and approved by the Ethics Committee of the joint research project “Participatory research to support sustainable land management on the Mahafaly Plateau in southwestern Madagacar (SuLaMa)”. The field research conducted in Madagascar was permitted by the Malagasy Ministry of Environment and Forests in collaboration with the World Wide Fund for Nature (WWF). As the study did not involve medicinal treatments, experiments, or the collection of personal data, it can be classified as being of minimal risk for study participants. Oral informed consent was given by the interviewees after receiving a short explanation about the goals and the procedures of the study by the researchers or market monitors. Obtaining written consent was not feasible since the majority of the population in the study region is illiterate. To ensure the practicability of the research under conditions of illiteracy of respondents, verbal consent was recorded in the protocols of market monitors by a checkbox.

### Data analyses

Data on the comic acceptance by local communities assessed through group discussions during the first survey was evaluated qualitatively (Table B in S1 Text) by identifying general pattern of the respondents’ answers and subjects for discussion that were noted by the moderator and the interviewers. Analysis were conducted through scanning primary data for words and phrases often used by the respondents to represent communities’ general point of view as accurate as possible, but in a way that generalizing statements could be made.

The majority of the data collected during the second survey included quantitative data (Table C in S1 Text). To facilitate data analysis, (response) codes were already defined in advance. Part of the data obtained during the second survey were collected qualitatively, but could be quantified due to their strong uniformity and often repetitive responses as specific questions were asked which offered little scope for comprehensive and thematically diverse answers by the respondents. This included a list of potential ecological or social consequences of continuing the current management practice or applying the recommended sustainable practice as well as challenges and opportunities for respondents of implementing the recommended practice. The quantification of these data was carried out by means of an abstraction and categorization of responses that followed the same meaning, but not the same wording. For instances, respondents’ perception of consequences regarding recommended practice on compost production and its application to crops included statements such as better plant performance, better yield, better plant health, and greener plants. All these statements followed the same meaning and were thus grouped into “Good plant performance”. However, special care was taken to ensure that the reasoning of the statements was not lost during quantification. Therefore, depending on the topic of the comic and the question, statements were categorized in different fineness. Using the example of potential positive effects of a sustainable wild yam harvest, a distinction was made as to whether the respondent expected a possible increase in yield or a continuous yield, since this plays a decisive role when it comes to the sustainability aspect of a management practice.

To analyze differences in the comprehension of local target groups based on the studied community and environmental subject of the comic we used linear mixed-effects models (LMER), considering respondent as a random grouping factor whereby some respondents were questioned about several comics.

To evaluate differences in the general understandability of comics among the studied communities, we calculated average age and average education level of respondents as well as the percentage of respondents who contributed with their own ideas about management improvements (INFEXT) and the percentage of respondents that already had an earlier encounter with either research projects or NGOs (PRENC; S1 Text). To obtain further descriptors for community structures, we made use of two indices (evenness and divergence) which are often used in community ecology science to describe the functional diversity of communities [48, 49]. Both indices, structural evenness (SEve) and structural divergence (SDiv), were explored by linking a respondent matrix with a respondent-socio-economic matrix considering gender, age, education level, INFEXT and PRENC as socio-economic individual parameters of a respondent. Structural evenness was measured directly by applying an evenness index to the abundance of respondents contained within each socio-economic category. In doing so, we received the SEve of a community that describes to which degree the number of respondents was equally distributed among the socio-economic parameters. The structural divergence describes the degree to which respondents’ socio-economic characteristics differ in their combination within a community and measures the variance of the respondents’ socio-economic status and the “position” within a community. We fitted linear models (LM) to assess the relationship between communities’ general comprehension and communities’ structure.

To gather individual-based effects on the comprehension of local people for the prepared comics, LMERs were calculated separately for each comic considering community as a random grouping factor. For each parameter that described comprehension and awareness, the full model, the null model and models with all valid combinations of the explanatory variables including first-order interactions were generated. To identify the relative support for each model, we used Akaike’s information criterion corrected for small sample sizes (AICc [50]). While the best fitting model in the set has the minimum AICc score, differences in the AICc scores between each model and the best model (Δ_i_ = AICc_i_—AICc_min_) and also the Akaike weights (w_i_) for each model were calculated. All models with ΔAICc < 2 entered the best model subset for model-averaged estimates (EMC) that were obtained by computing means and adjusted standard errors of the estimates weighted by the model weights. This approach quantifies a relative variable importance (RVI) that suggests the relative contribution of each explanatory variable in explaining the variance in the response variable [51]. Because of data imbalance, gender as explanatory variable was excluded from those models that were used to extract effects on the peoples’ comprehension and awareness for the “samata” comic, which was basically addressed to male herders and cattle owners [52].

The third survey, which was conducted with the aim to evaluate to what extent the recommended practice was implemented by the interviewees over the past months, contained both quantitative (categorical and binary) and qualitative data (Table D in S1 Text). Since here recurring answers with similar meanings were given during the interviews, responses could be reduced and grouped to a few key words, wherefore these data could partly be evaluated quantitatively. In contrast to the topic of “samata” utilization and wild yam harvest, both activities in which all interviewed community members were involved, data that derived from interviews that focused on compost production and the willingness of respondents to adopt the recommended practice was limited to respondents who owned livestock. However, only eight out of 112 respondents were not involved in livestock husbandry and thus data evaluation was not significantly affected due to minor data exclusion.

All statistical analyses were conducted in R version 3.3.0 [53] with additional functions provided by the R packages FD [54, 55], lme4 [56] and MuMIn [57].

## Results

### Comic acceptance by local communities assessed through group discussions

The graphic implementation of the environment, especially the drawing of zebu cattle, found high interest among all participants of the group discussions and became the first important point of debate. Discussion groups quickly recognized that both illustrated stories within one comic have the same topic but different contents and propositions. The “red story”, illustrating practices and consequences of a common, but unsustainable management practice, was perceived consistently across all groups as a bad and sorrowful process. The “green story” in contrast, perceived appreciation mainly due to its healthier appearance of plant and animals. Particularly the materials that were used by the depicted people as such as the knife to cut “samata” (“samata” comic), the can for watering papaya or the cooking stove (both in the compost comic) received much attention. Groups further discussed comprehensively the details of the story such as the drunken man (compost comic) and even implied that children in the “green story” attend school (compost and “samata” comics), although this was not intended to be meaningful.

### Communities’ normal practices and understanding of the comic

Interviewees’ response on the question whether their normal practice of management in our target topics was closer to the bad scenario or to the recommended varied among the studied communities (ANOVA, *F*_140,5_ = 3.2, *p* ≤ 0.05). It was highly dependent on the topic of the presented comic (ANOVA, *F*_128,2_ = 54, *p* ≤ 0.001). The majority of the respondents indicated, as to be viewed in Table 1, that they already used the recommended practice of composting and fertilization. Almost all respondents stated to use “samata” sustainably as recommended within the comic. In contrast, wild yam harvest was admitted by the large majority of respondents to be practiced as illustrated in the unsustainable story.

**Table 1:** Normal practice of respondents in six communities of the Mahafaly region in southwestern Madagascar.

Practice	Compost production		“samata” utilization		Wild yam harvest	
Unsustainable	29%	(n = 30)	2%	(n = 1)	90%	(n = 52)
Improved	9%	(n = 9)	0%		0%	
Recommended	62%	(n = 65)	98%	(n = 40)	10%	(n = 6)

Percentage and number (in bracket) of respondents

**Fig 3:**
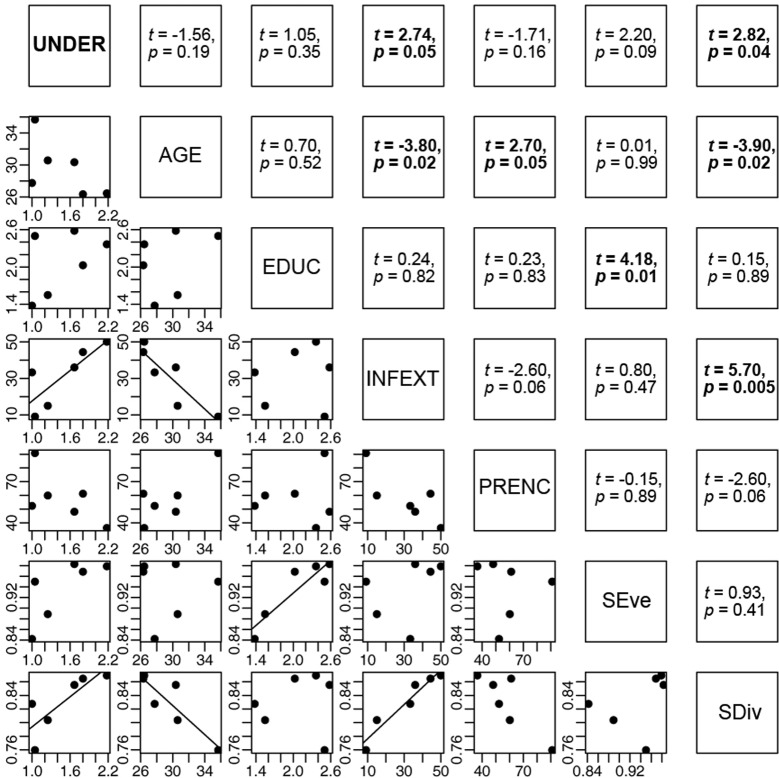
Relationship between average comic understandability (UNDER) of a community and communities’ structure. Communities’ structure as described by mean age (AGE), mean education level (EDUC), percentage of respondents with inferences for extension (INFEXT), percentage of respondents with prior encounter to research projects and/or NGOs (PRENC) as well as descriptors of communities’ structure as evenness (SEve) and divergence (SDiv). The data refer to six villages in the Mahafaly region of southwestern Madagascar.

The general understandability of comics strongly depended on the surveyed community (ANOVA, *F*_155,5_ = 5.8, *p* ≤ 0.001) and on the topic of the comic (ANOVA, *F*_141,2_ = 3.6, *p* ≤ 0.05). The “samata” comic was best understood by the respondents (LMER, *t*_212_ = 4.9, *p* ≤ 0.001) whereas the wild yam comic seemed difficult to understand (LMER, *t*_103_ = -2.1, *p* ≤ 0.05). Communities with an increased proportion of members who contributed their own ideas for improving land management strategies (INFEXT) were more at ease with the content of the comics than other communities (LM, *t*_4_ = 2.74, *p* ≤ 0.05; Fig 3). We further found a positive correlation between the general understanding of the comic and the divergence in communities’ socio-economic structure (LM, *t*_4_ = 2.82, *p* ≤ 0.05). Models further revealed that an increase in mean age of the respondent group was correlated with a mitigation of extremes found in respondents’ socio-economic characteristics (SDiv). Mean age or mean education level of a community was not directly related to a better understanding of the prepared comics, but showed strong relationships to other community structural parameters. For instances, a respondent group with a high proportion of members that finished primary school showed in general a more even distribution of members (LM, *t*_4_ = 4.18, *p* ≤ 0.01) among different socio-economic parameters as such as among age classes and education levels.

### Individuals’ comprehension and awareness

Results from individual based interviews revealed that respondents who keep livestock in corrals have a better understanding of the comic focusing on composting manure and organic HH waste than respondents without livestock, regardless of whether they already followed the recommended strategy (EMC = 0.8 ± 0.3, *p* ≤ 0.01) or not (EMC = 1.0 ± 0.3, *p* ≤ 0.01). Results further revealed a positive correlation between education level and general understanding (EMC = 0.1 ± 0.06, *p* ≤ 0.01). Both parameters, normal practice and education level, were also positively correlated with the ability to quickly recognize differences between the two illustrated practices (unsustainable *versus* recommended) as well as with the ability to assess environmental consequences (awareness) of the opposing practices. The ability to conclude that this comic intended to illustrate two stories was less pronounced in men than in women (EMC = -4.7 ± 1.8, *p* ≤ 0.01), which increased with the age of men (EMC = 0.13 ± 0.05, *p* ≤ 0.05).

In contrast, respondents’ understanding of comics illustrating a sustainable practice for “samata” utilization or wild yam harvest could be explained only to a small extent by our explanatory variables, namely only by PRENC and/or INFEXT that strengthened overall comprehension and awareness of respondents. Other variables as such as respondents’ gender, age or education level were only poor predictors for their comprehension and awareness of the illustrated management practices.

### Challenges and opportunities of SLM practices and readiness to act

The data summarized in Table 2 shows that the majority of respondents stated to see no problems in implementing the recommended practice of composting manure and organic HH waste and harvesting “samata” and wild yam. Nevertheless, water availability was mentioned frequently by farmers to be a challenge in implementing the recommended practice. The main advantage of co-composting of manure with organic HH waste and its application to crops was the perceived increase in yield and with it HH income and food security. Food supply and food security was also mentioned by the majority of interviewed locals when evaluating opportunities that a sustainable wild yam harvest can offer. Fodder availability that enhances livestock production was mentioned as a key opportunity that can be attained by a cautious removal of the coral-like spinous branchlets when harvesting “samata”.

**Table 2:** Respondents’ observations on normal practices and their perception on challenges and opportunities in implementing the recommended practice.

Compost production Percentage of respondents (%)		”samata” utilization Percentage of respondents (%)		Wild yam harvest Percentage of respondents (%)	
**Respondents’ perception on challenges in implementing recommended practice**					
None	57%	None	73%	None	55%
Water	20%	Water	10%	Soil recover	15%
Seeds	11%	Protection	7%	Water	12%
Manure	5%	Land	7%	Missing technique experience	9%
Time	3%	Time	3%	People	6%
Protection	2%			Plant resource	3%
Climate	2%				
**Respondents’ perception on opportunities through implementing recommended practice (RP)**					
Increased yield	57%	Fodder availability	55%	Food supply & Food security	92%
Finances	26%	Livestock production	21%	Increased yield	8%
Food supply & Food security	14%	Finances	12%		
Seeds	3%	”samata” resources	9%		
		Reduced collection distance	3%		
**Percentage of respondents observing the recommended practice in their immediate environment**					
	83%		100%		24%
*Which persons were observed that used the recommended practice (RP)*?					
Family members	60%	Family members	70%	Neighbor	50%
Neighbor	15%	Villager	18%	Family	38%
Villager	15%	Neighbor	9%	Villager	12%
Friends	10%	Friends	3%		
**Percentage of respondents observing unsustainable practice in their immediate environment**					
	56%		88%		90%
*Which persons were observed that used the unsustainable practice*?					
Family	33%	Foreigner	62%	Friends	37%
Neighbor	33%	Neighbor	18%	Villager	23%
Friends	24%	Villager	14%	Family	20%
Villager	10%	Family	3%	Neighbor	14%
		Friends	3%	Foreigner	6%
*Spontaneous action of respondent to this observation*					
Nothing	59%	Advised RP	52%	Nothing	84%
Advised SP	41%	Nothing	28%	Advised RP	10%
		Informed	20%	Informed	6%
**Percentage of respondents who shared the newly acquired knowledge with their social environment**					
	60%		70%		66%
*Who was the main addressee*?					
Family	60%	Friends	50%	Family	74%
Friends	35%	Family	25%	Friends	13%
Neighbors	5%	Neighbors	25%	Neighbors	13%
**Reason of respondents for not sharing the acquired knowledge with their social environment**					
No occasion	42%	No occasion	50%	No occasion	33%
No need	25%	No need	38%	No need	33%
No time	21%	No interest	12%	No time	25%
No interest	12%			No understanding	9%

Percentage of respondents that were interviewed within six communities of the Mahafaly region in southwestern Madagascar, 2015.

Although 98% of the respondents stated to harvest “samata” by applying the recommended practice, 88% also confirmed to have seen foreigners from neighboring villages or itinerant cattle traders to poll these plants destructively. Destructive harvesting of wild yam was also very often observed by interviewees of whom the majority admitted not to have acted by informing or advising the observed person about alternative, sustainable practices. The technique of co-composting manure and organic HH waste had been observed by a high proportion of respondents who further reported that primarily family members were observed to apply this practice. Between 60 and 70% of the interviewed farmers had shared the comic with family and friends. The most common reasons for not passing the comic to relatives, friends and/or neighbors were either missing occasion (33–42%) or because there was no perceived need for it (25–38%).

### Willingness of adoption

The willingness of respondents to adopt the recommended practice for harvesting wild yam was weaker than their willingness to broaden the usage of manure (LMER, *t*_34_ = -2.2, *p* ≤ 0.05). Nevertheless, 75% of the interviewed locals showed a willingness to implement the recommended practice for harvesting wild yam. Almost all interviewees (96%) that did not use a mixture of manure and organic HH waste for compost production and subsequently as fertilizer declared to adopt the recommended practice.

However, the willingness of respondents to follow the recommended practice of wild yam harvest was impaired when respondent saw no change in the improved technique compared with the common one (LMER, *t*_39_ = -2.9, *p* ≤ 0.01). Although not statistically significant, a weak willingness of adaptation was accompanied with lower education level (LMER, *t*_40_ = -1.8, *p* = 0.074) and with respondents’ opinion that the current practice has no consequences on future wild yam population (LMER, *t*_39_ = -2.0, *p* = 0.052) as shown in Table 3. Respondents’ perception that the current practice does not have any consequences (LMER, *t*_32_ = -2.2, *p* ≤ 0.05) or that the recommended practice does not bring any change (LMER, *t*_31_ = -2.5, *p* ≤ 0.05) impaired also the willingness to adapt the technique of co-composting manure and organic HH waste.

**Table 3:** Respondents’ perception of consequences regarding unsustainable and recommended practice.

	Compost production		Wild yam harvest	
*Respondents’ perception of consequences regarding unsustainable practice*				
	Bad plant performance	78%	No consequences	28%
	No consequences	18%	Insufficient yield	23%
	No response	4%	Plant extinction	18%
			No yield	14%
			Reduced plant regeneration	10%
			No response	7%
*Respondents’ perception of consequences regarding recommended practice*				
	Good plant performance	78%	Increased yield	35%
	No changes	18%	Continuous yield	32%
	No response	4%	No changes	21%
			Improved plant regeneration	12%

Percentage of respondents that were interviewed within six communities of the Mahafaly region in southwestern Madagascar, 2015.

Results from the third survey revealed that only a small proportion of respondents (eight out of forty) stated to have collected wild yam during the last weeks. Hence, the subset of data that was used as reference to compare the declared willingness of adoption by the survey participants with a factual implementation of the recommended practice did not provide a sufficient basis for evaluation. Nevertheless, three out of eight respondents stated to have changed their normal practice and adapted the recommended strategy for wild yam harvest. Furthermore, all five respondents who collected wild yam following their traditional practice admitted that the sustainable harvest technique as illustrated within the comic was not completely understood wherefore the recommended practice was not applied. For compost production, only a small proportion of respondents (nine out of 75) stated to have produced compost during the last weeks wherefore only a small subset could be evaluated. However, seven out of nine respondents affirmed to have adopted the recommended practice.

### Comic review through respondents and interviewers

During the surveys, interviewers repeatedly remarked that many respondents had difficulties putting the sequence of the images on a temporal scale, as presented in Table 4. The symbolization of HH wealth and income security through the use of a more ornate and pompous “aloalo” was also not recognized as such by many respondents. Great importance was attached to the correct shape, posture and color of animals and plants, which is why many respondents repeatedly pointed to a faulty representation of the reality in the comics.

**Table 4:** Recurring comments by respondents and interviewer to the structure and content of comics.

Compost production	“samata” utilization	Wild yam harvest
• Often difficulties in linking time and/or season with the sequence of the pictures in the comic • Often difficulties in relating “aloalo” structure (simple *versus* ornate) to core messages of scenarios		
• Atypical posture of zebu (grazing) within corral • Too little difference between underfed and well-fed zebu Drunk man is often seen as dead body	• Difficulties in recognizing the indicated rainy season (rain) • Men often failed to identify with “crying man” as frank emotions are not part of the Madagascan culture	
	• Cemented house as residence looks atypical • Atypical color of zebu (felt to be too dark)	• Atypical method of digging a hole (hunched over) • Atypical color of branches (felt to be too green) • Recognized rain as smoke

## Discussion

### Use of silent comics for scientific knowledge transfer and development cooperation

For a long time, silent comics and illustrations are considered suitable tools for knowledge transfer of scientific results and communication for development to the rural poor and often low-literate communities in developing countries [28, 58]. They are regularly used to impart knowledge on new agricultural technologies and innovations as well as on environmental and health aspects. They go along with other communication media such as videos [22, 23], theatre performances [59, 60] and role-playing games [61, 62]. Nowadays, a stronger focus is given to new information and communication technologies (ICT) based on the use of mobile phones and the internet in addition or exchange to traditional media and extension services, especially in remote rural areas [63, 64]. In this context, Maredia et al. [65] described the effectiveness of delivering information about new agricultural technologies to low-literate farmers in Burkina Faso through mobile-phone-based animated videos. The widespread availability of new ICTs in rural areas of developing countries offers new opportunities, but still faces considerable limitations through incomplete mobile phone coverages and slow internet connections [24].

In the Mahafaly region, mobile-phone coverage only spread out very recently together with the availability of mobile internet. It was still in its infancy when the data collection for this study on graphic narratives took place and the vast majority of local residents then only owned old-fashioned mobile phones with no or limited internet capability, if they owned a phone at all. ICT-based visual communication tools such as videos and digital illustrations may therefore not yet be easily accessible on a broader scale for the target groups for whom they are intended. Furthermore, the conscious handling of such digital applications by people who only discovered the usage of internet-ready devices a short while ago and often are not used to complex digital media without previous and comprehensive instructions may be difficult and finally go at the expense of understandability and implementation. Under such conditions, the use of new ICTs may appear more futile and communication of scientific outcome to the local communities in form of “traditional” visualization tools such as silent comics can still be seen as an appropriated instrument for knowledge transfer and communication for development, if distributed in a purposeful manner.

Besides the usage of comics, Wesselow and Stoll-Kleemann [66], for example, reported on the implementation of a participatory role-playing game, which dealt with the maintenance of livelihoods for local households in the Mahafaly region. Following the results of non-systematic interviews with participants, they concluded that the game well functioned as innovative communication tool in natural resource research and management that induced changes in the players’ livelihood decisions, but also indicated that the learning was taken further into the community. However, for the participatory role-playing game Wesselow and Stoll-Kleemann [66] used further visualization tools and attached great importance to them in helping to translate and understand the perceptions of the various participants and to reach a common understanding beyond language barriers.

Other projects and national as well as international NGOs active in the area, such as the World Wide Fund for Nature (WWF), rely on film screening and environmental comics to increase environmental awareness among the local population and in particular school children (Feldt, personal observation). These actions, however, often lack a retrospective and systematic appraisal of their sustainable impact on the targeted communities. In our study, this common weak point was encountered by the three-step approach to evaluate the first acceptance of the comics immediately after their distribution as well as their understandability and possible implementation with a time lag.

### Individuals’ awareness for addressed SLM practices

When most of one’s way of life is built on habits and one is unaware of the consequences one’s behavior will cause, than it does not really matter if those acts lead to environmental problems or not [17]. Norman [67] argues that stories are needed to cope with everyday life as stories provide access to the reality more than logic. The central aim of our approach using graphic narratives in the style of a silent comic was to increase the environmental awareness of local communities and to provide recommendations on SLM in the Mahafaly region of southwestern Madagascar. Linking land management strategies with natural resources and livelihood is, however, very complex as cultural, socio-economic and environmental drivers of change are intimately linked [68]. The combined method of qualitative data of group discussions and quantitative data of semi-structured individual-based interviews that built upon each other was helpful to evaluate peoples’ understanding of SLM at the community and individual level.

The understanding of our comics was highly dependent on the illustrated environmental topic and was influenced by respondents’ practice. With regard to the comic on compost production, Bayala et al. [69] and Coral Guerra [37] found that the majority of farmers in the Mahafaly region do not use livestock manure for field fertilization. The reasons given by farmers were the high labor demands for manure application, increased disease pressure, lack of knowledge for its use and the perceived relative fertility of the soil [11, 69]. Farmers prefer to shift crop fields to new land if yields are declining, instead of investing in labor-intensive fertility management [70]. Manure, abundantly available in village livestock corrals, seems to be of too low quality with decreased decomposition rates and small availability of plant nutrients due to ammonia volatilization in the hot dry season, losses through nitrate leaching and denitrification in the rainy season as well as organic matter that might be rather recalcitrant due to its long storage [10]. At the same time, the widespread practice of parking animals closely into relatively small enclosures at night, while these are hardly ever cleaned from the animals’ feces, increases risks of epizootic diseases and parasite infections, both found frequently within the region and affect livestock severely [12, 38]. Hence the comic about compost production was thematically very complex and intended to communicate three recommendations, namely regular corral cleaning (livestock health), production of compost using manure and organic HH waste (resource usage) and the application of water and compost to home-grown crops (soil fertilization). While asking for respondents’ normal practice, however, questionnaires did not consider a separation of the three different recommendations. Thus an affirmative answer of respondents that might include only one practice of three could result in misleading conclusions.

Nevertheless, education level and normal practice were two determining factors that positively influenced respondents’ understanding regarding the preparation of co-composting manure and organic HH waste (Table E in S3 Text). Particularly in recognizing differences between unsustainable and sustainable practices as well as in assessing the benefit of applying the recommended practices, higher educated respondents seemed to be advantaged. Against the background that in many developing countries the majority of farmers have not received any schooling and are hence illiterate, it is easy to imagine that lack of education is a severe obstacle for farmers when changing their traditional land management strategies [71].

The graphical implementation of consequences of an unsustainable and an alternative, sustainable practice for “samata” utilization was well understood by the majority of respondents as their current practice was stated to consider a careful and only partial removal of branches. Indeed, in many communities of southwestern Madagascar, rules for a sustainable harvest are traditionally defined even if a disregard is not always pursued. In recent years, however, unregulated privatization of former communal land has gained importance [41], which leads to unequal access to “samata” as fodder resource and consequently to an overuse of freely available stands of *Euphorbia stenoclada*. In particular non-residents were observed to excessively remove branches. At present, for socio-cultural and practical reasons, municipalities do not restrict resource use by foreigners [41]. This makes it all the more important that half of the respondents stated that they had approached outside violators in order to inform and advise the person being observed about a resource-conserving use of “samata”. *Euphorbia stenoclada* is an important dry season ruminant fodder for the livestock-dependent inhabitants of Madagascar’s southwestern region [39, 40] and local communities have already made an effort to raise awareness of “samata” resource depletion. Therefore we could probably not find a relationship between respondents’ socio-economic characteristics and their comprehension and awareness of the comic.

For respondents’ understanding of the wild yam comic, socio-economic parameters as such as age, education level or prior encounter with research projects or NGOs could also not serve as reliable explanatory variables. In contrast to the environmental concern about “samata” scarcity among local communities, the problem of over-exploitation of wild yam, leading meanwhile to an alarmingly low abundance of wild yam species in the Mahafaly region [72], is still neglected and not understood as a consequence of destructive harvests. Nevertheless, more than 90% of the respondents recognized the opportunity of having a steady food supply and with it food security when harvesting sustainably wild yam plants and one-third of the respondents stated to have already observed neighbors harvesting yams sustainably. Although wild yam harvest used by most respondents was destructive, overall willingness of respondents to adopt the recommended practice of wild yam harvest was high. All respondents who applied the new technique stated to have observed it with others, whereas respondents, who did not apply the recommended technique consistently stated that they have not observed this method in their social environment during the past weeks and that the recommended technique illustrated within the comic was not completely understood. Social networks among farmers have been shown to be an important factor when changing agricultural practices particularly when they are on-the-ground and observable by neighbors [73–75]. Truelove et al. [76] also noted that farmers in Sri Lanka, who interact with others, especially with his/her community, are more likely to adapt new agricultural practices.

### Communities’ awareness for addressed SLM practices

When scaling up respondents’ general understanding of comics illustrating common practice and recommended SLM practice at the community level, we found a strong relationship between the general understanding and the divergence in communities’ socio-economic structure. Structural divergence of communities comprised the interrelation between age, gender, education level, INFEXT and PRENC. For a single socio-economic approach, structural divergence represents how respondents are spread in numbers along a parameter axis within the range that exist within the community [48, 49]. For instance, structural age divergence is low when respondents are of similar age. Conversely, when many respondents are of “extreme” age, then structural age divergence is expected to be high. On average, highest education level that in our study area meant finishing primary school was found for the interviewed groups in the villages Andremba and Ankilibory. This, however, was not connected to an overall better understanding of the comics. Respondent groups of the Efoetse community showed best understanding of the presented comics and in comparison to the interviewed communities of Ampotaka, Marofijery and Miarintsoa also a similar education level to that of Andremba and Ankilibory. In contrast, Efoetse was further characterized by the highest percentage of respondents who developed during interviews their own ideas for improved practices but also the lowest percentage of respondents who declared to have no former contact to other research projects and NGOs. Franz et al. [77] concluded that some focus group participants do not value information and resources from agents that are often not experienced with their agribusiness. Therefore prior encounters with NGOs can have a negative impact on adoption of new practices or techniques communicated through cooperation with projects and extension workers. Farmers want a one-to-one attention on their farm as well as agents who provide information and techniques they really need and are able to implement [77]. Hence, irrespective of education level, communities in which members show good extension skills for recommended SLM practices processed by scientists are expected to implement them successfully to cope with future climate challenges. A further crucial factor was mentioned by Esham and Garforth [78], namely that farmers who more often communicate the information about climate change and adaptation measures with others adopt innovations by themselves. Although a high percentage of respondents stated to have shared the comic, the transmission and discussion, however, took place primarily in their close social environment (family and friends) and only to a small extent with neighboring farmers.

### Respondents’ willingness of adoption

The education level of respondents affected respondents’ willingness of adoption. Reimers and Klasen [71] revisited the role of education for agricultural productivity and concluded that primary and secondary education has a significantly positive effect on agricultural productivity that is coupled with the willingness of farmers for adoption of innovations, which can be strengthened due to enhanced decision-making skills. Better educated farmers adopt new practices faster as they are not only capable of using information more competently; they also have better access to the required information [79]. On the other hand, Fraslin [80] argued that most education in rural Madagascar is not geared toward agricultural knowledge and is therefore of little direct use to farmers [15]. Furthermore, the effect of education on adoption rates is generally smaller in countries with still traditional agricultural settings and slow technical change [71, 81, 82]. We therefore presumably missed strong effects of education on overall respondents’ awareness and willingness of adoption for improved “samata” utilization and wild yam harvest as particularly in the Mahafaly area traditional agriculture predominates. Here, fragmented production, low productivity, overuse of natural resources, vulnerability to natural hazards and limited access to economic and commercial opportunities due to isolation, in addition to the political crisis of 2009–2013, hampered socioeconomic and in particular agricultural development and lead to an increase in rural poverty. Potential effects of education level on respondents’ motivation for final implementation of the recommended practice could not be clarified in the framework of this study as only a small subset of data was available. However, a study of Fisher [83] about demographic effects on adoption rates of *Brachiaria* grass cultivation by Mahafaly zebu breeders revealed that predominantly younger, more educated, and more mobile breeders decided to adopt the new technique.

The most mentioned challenges for the adoption of the illustrated SLM practices by the respondents were water shortage, limited seeds availability but also missing knowledge on proper techniques, particularly regarding the sustainable harvest method for yam. High labor demands, lack of transport and materials as well as inadequate knowledge and small social capital are commonly cited factors that impede adoption initiatives of sustainable practices to increase agricultural productivity in rural Madagascar [13, 15, 84–86]. Furthermore, in the context of adoption decisions and rates of sustainable agricultural practices in African countries, differences in gender become increasingly important. Numerous studies confirm the need for a more nuanced gender data collection and analysis as it is crucial for designing effective policies and training manuals to close the gender gap [87–91]. This is important as African women play a key role in agricultural development where they are responsible for family food security and home production [92].

### On the effect of style and visualization to raise awareness of silent comic illustrations

Since cross-cultural differences arise in “visual languages” globally, the visualization style needs to be carefully selected. Misunderstanding of visual narratives easily occur when objects are drawn not realistically enough, or if the statement is abstract or only presented from a scientific point of view [28, 29]. Fuglesang [18] concluded that learning to read pictures is analogous to learning to write a language that in turn depends on the educational environment. Rural villagers in developing countries, particularly in Africa, often lack any schooling wherefore the interviewed communities had difficulties in recognizing the time component of the two SLM scenarios that was indicated through the sequential flow of pictures (left to right) but also through the drawing of rain, indicating the rainy season. As the sequence of a story is most often causal, missing skills in reading pictures in correct order can hamper the understanding of SLM recommendations [93] that weaken farmers’ willingness of adoption.

An atypical representation of zebus and plants was often commented by the respondents although here great importance was attached by the designer to a realistic reproduction in shape and color. Hence, if color is one essential aspect of a graphic narrative that is intended to communicate information in a cultural context, especially among populations with no formal education, materials should undergo several testing rounds with an appropriate cross section of the target population beforehand. Otherwise they may fail to transmit the information they are supposed to [20, 94]. Stories told through silent comics can be a useful communication tool to increase awareness and comprehension in illiterates and children for SLM practices as they transfer socially and playfully information, knowledge, context, and emotion [67]. However, details in graphic narratives like the drunken man who was considered dead by many respondents or the cemented house, which should symbolize prosperity but seemed not to correspond to local architecture of a private residence presumably due to a dissonance in color scheme and the illustration of two entrances, can apparently negatively affect the central message. Hence, pictorial elements that are needed to personalize a story and that are used to indicate either consequences or opportunities of SLM practices should only be used on the basis of well-founded local observations to avoid distraction by unnecessary "side stories" [20, 28, 95]. Nevertheless, although particularly male respondents repeatedly stated that crying is not part of a man’s openly shown behavior, except at funerals, wherefore they had difficulties in seeing themselves reflected in this story, the drawing of the crying man, however, gave the story an emotional touch. Davidson et al. [96] and Adler [32] found that we remember more emotional parts of a story than non-emotional ones, which may have something to do with our memories being interconnected to such contents [17]. When taking part in a story we adopt a point-of-view situated inside the story world. The closer our point-of-view is to the main character in the story the faster we will recall and the more affected we will become afterwards [17, 97].

## Conclusions

The creation of silent comics that intend to communicate recommendations on sustainable land management practices is a challenge but also an opportunity for development projects. This study could demonstrate that visual communication tools should be thoroughly tested in the region of application by using a multi-level, successive approach that considers the perspective of all actors (drawing artists, local population, conducting interviewers and multidisciplinary scientific experts) before being put to use for educational purposes in cross-cultural contexts. It may transfer knowledge where oral communication fails because of linguistic barriers or simply because complex narratives and messages are easier to explain visually than orally. Silent comics can be also a useful communication tool when it comes to initiate a vital dialogue between science and society and to raise environmental awareness.

However, pictorial communication tools are far from self-explanatory and the transfer of complex information, as it occured with our comic on recommendations for compost production, needs further explanatory elements, such as a written instruction manual. Hence, our study once more highlighted that formal education and social environment alone has a strong effect on peoples’ comprehension and awareness of interdependent natural processes and on their willingness for adoption of SLM practices. However, as communities faced further limiting factors such as overall low literacy rates and limited water, land and seed resources, the real impact of education, whether formal, non-formal or hands-on-training, might be partially masked within the framework of our study. In order to improve the awareness, comprehension and motivation of local communities for SLM practices, there is a need to consider communities’ heterogeneity in age, level of education, extension services outreach, and socio-economic characteristics.

Pictorial communication tools are far from self-explanatory as style and iconic significance often convey culture-specific as in our case for people of the Mahafaly region in southwestern Madagascar. Hence, although comics’ easy reproduction and distribution clearly has an advantage over oral communication, graphical narratives, particularly with culture-specific drawing details that are used to facilitate farmers’ ability to adopt a point-of-view situated inside the comic story, have a regionally limited impact and are therefore not universally applicable. Our study further revealed that culture-specific drawing details must be used thoughtfully as they might interfere with the central message.

Nevertheless, the explanatory power of the three silent comics went beyond the evaluation of locals’ understanding regarding the content of graphic narratives that intend to encourage people to implement new sustainable management practices in their daily life. Questions on respondents’ awareness of consequences and perception about challenges in the implementation of SLM strategies allowed a bi-directional knowledge transfer that, in turn, is fundamental if organizations involved in agricultural extension and policy makers are to support rural populations in using landscapes sustainably to foster environmental health and productivity.

## Supporting information

S1 Fig Image of comic about compost production–“red story”. (TIF)

S2 Fig Image of comic about compost production–“green story”. (TIF)

S3 Fig Image of comic about “samata” utilization–“red story”. (TIF)

S4 Fig Image of comic about “samata” utilization–“green story”. (TIF)

S5 Fig Image of comic about sustainable wild yams harvest–“red story”. (TIF)

S6 Fig Image of comic about sustainable wild yams harvest–“green story”. (TIF)

S1 Text Materials and methods—Number of conducted interviews within the six communities and additional information on qualitative data analysis with posed questions and collected data types. File consists of Tables A-D.
(PDF)

S2 Text Materials and methods—Copy of survey and interview guideline in French and English. (PDF)

S3 Text Results—Factors influencing positively or negatively the comprehension and awareness of respondents in six communities of the Mahafaly region in southwestern Madagascar. File consists of Table E.
(PDF)

S4 Text Data set–Data collected during individual based interviews (second and third survey) within the six communities. File consists of Excel spreadsheets.
(XLSX)
