# Supplementary figures and images for: The explanatory power of silent comics: An assessment in the context of knowledge transfer and agricultural extension to rural communities in southwestern Madagascar
Source: PLoS One. 2019 Jun 6;14(6):e0217843. doi: 10.1371/journal.pone.0217843 (PMC6553738; doi:10.1371/journal.pone.0217843)

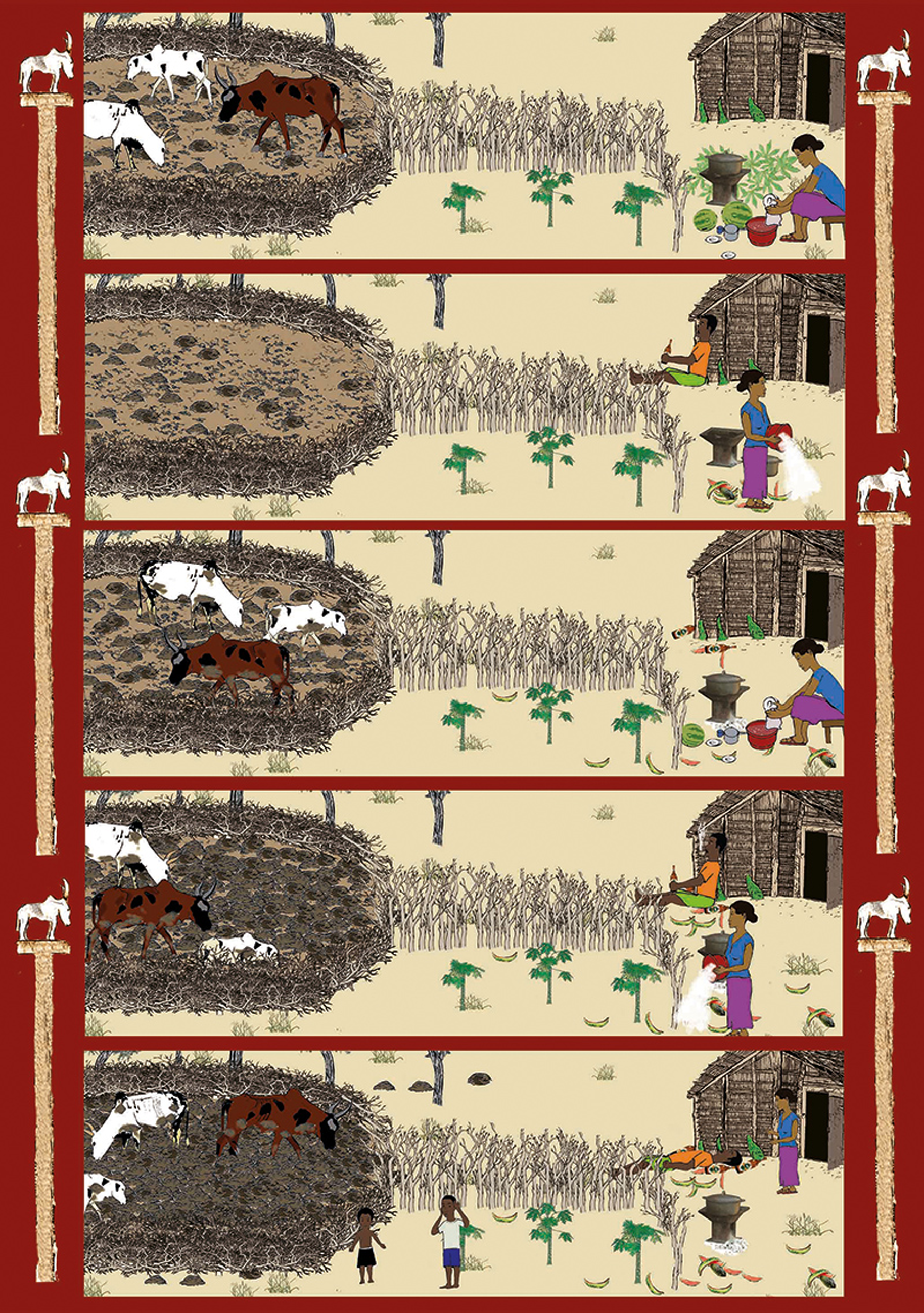

Supplement: S1 Fig — (TIF) [file pone.0217843.s001.tif]

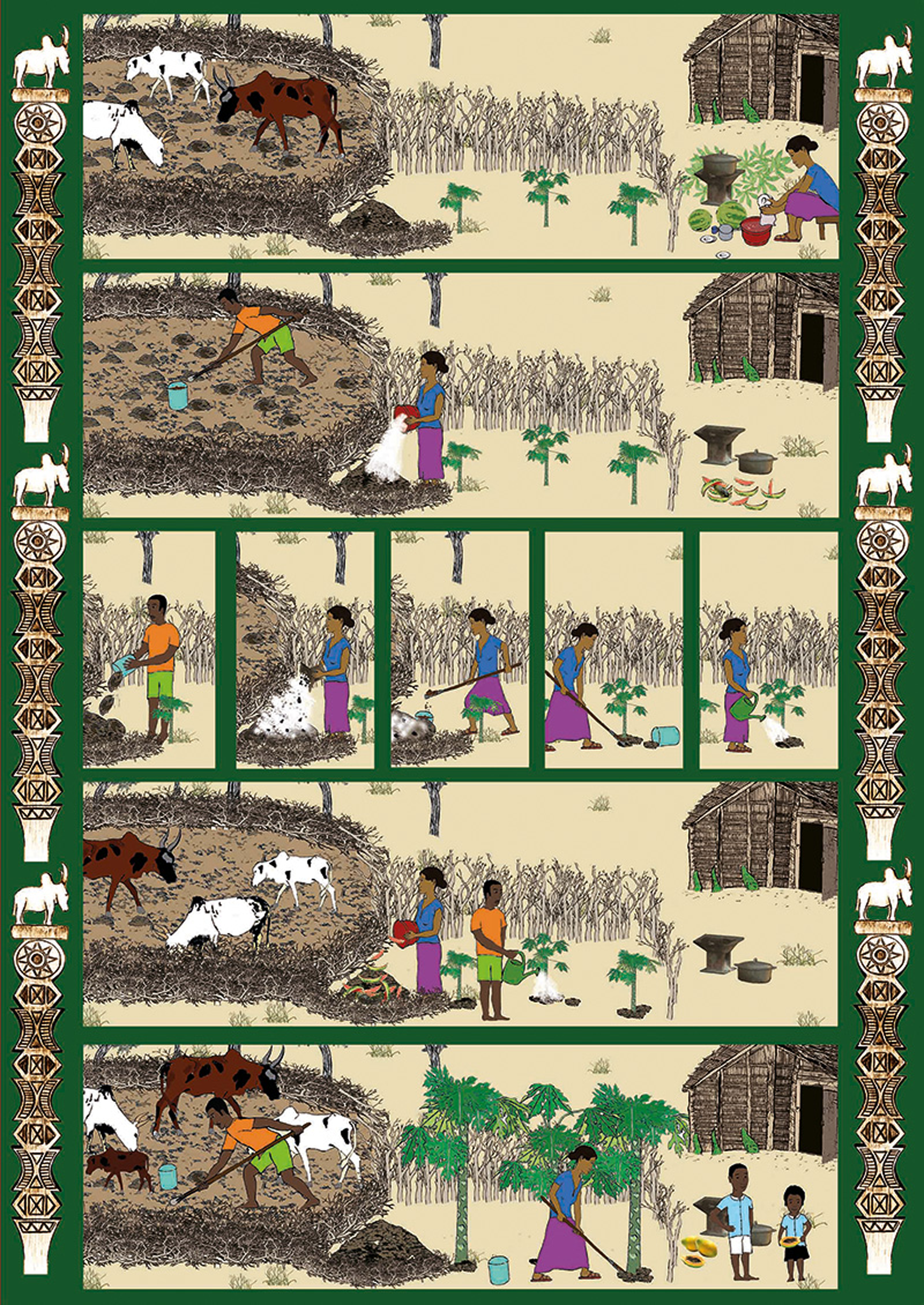

Supplement: S2 Fig — (TIF) [file pone.0217843.s002.tif]

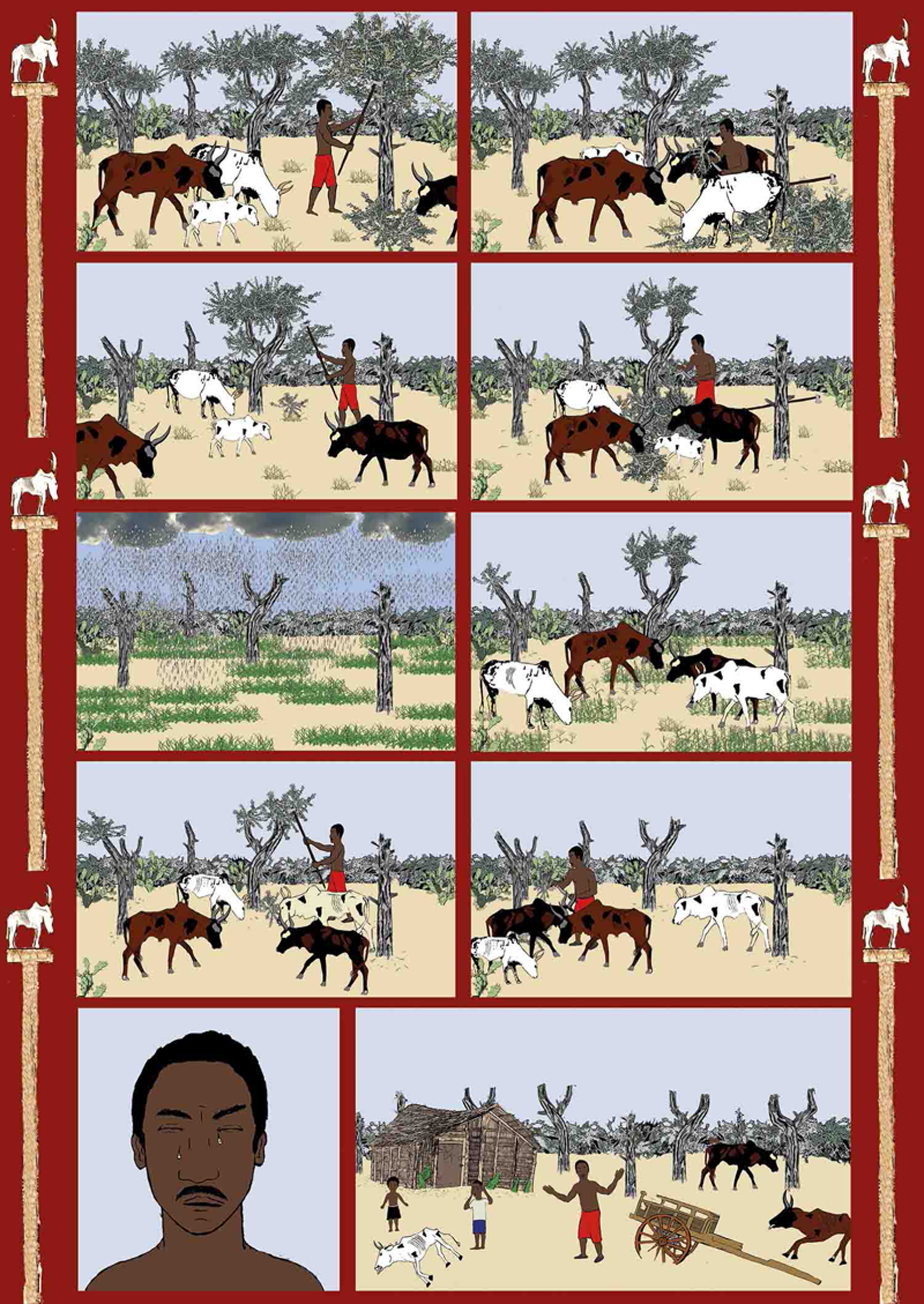

Supplement: S3 Fig — (TIF) [file pone.0217843.s003.tif]

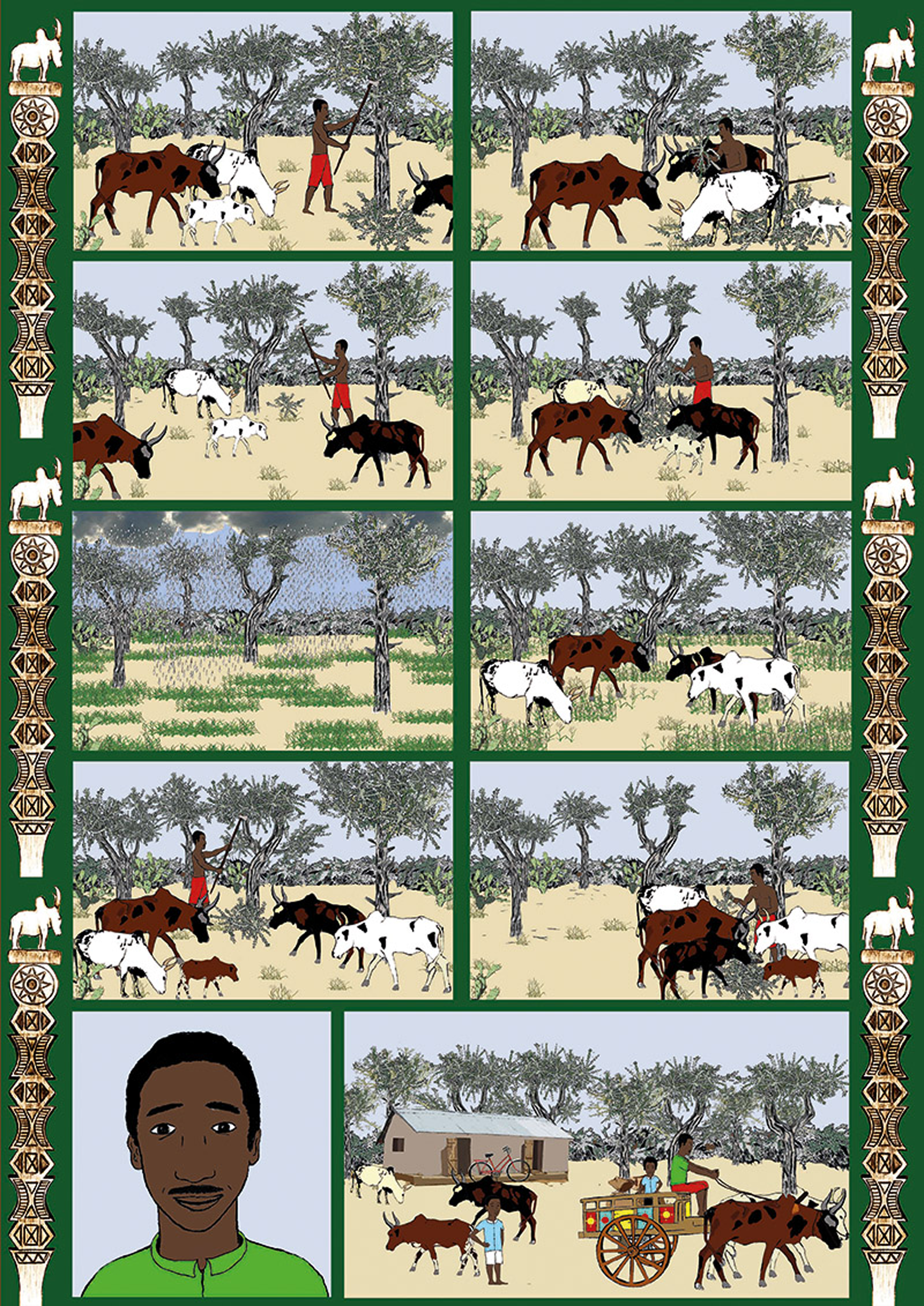

Supplement: S4 Fig — (TIF) [file pone.0217843.s004.tif]

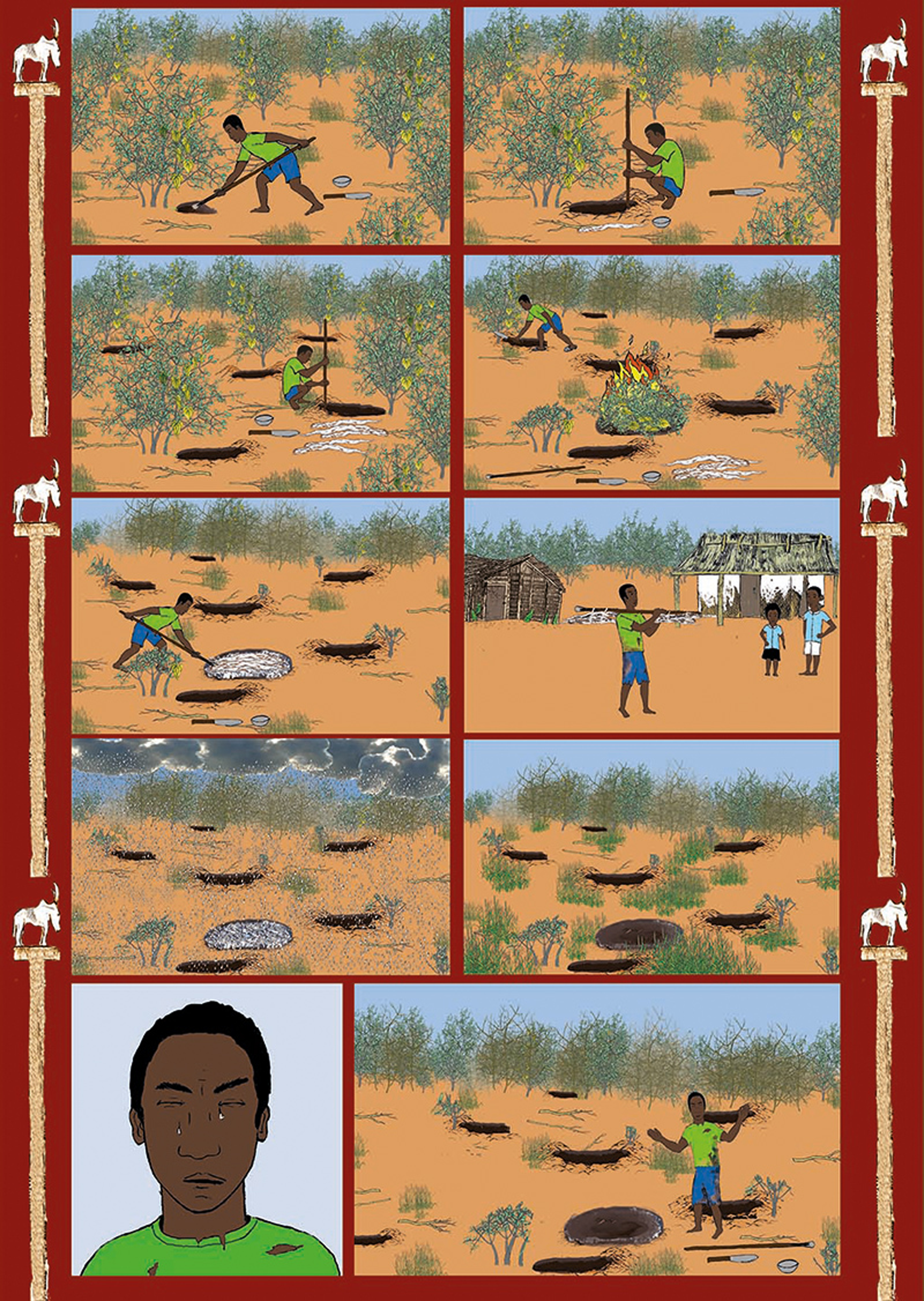

Supplement: S5 Fig — (TIF) [file pone.0217843.s005.tif]

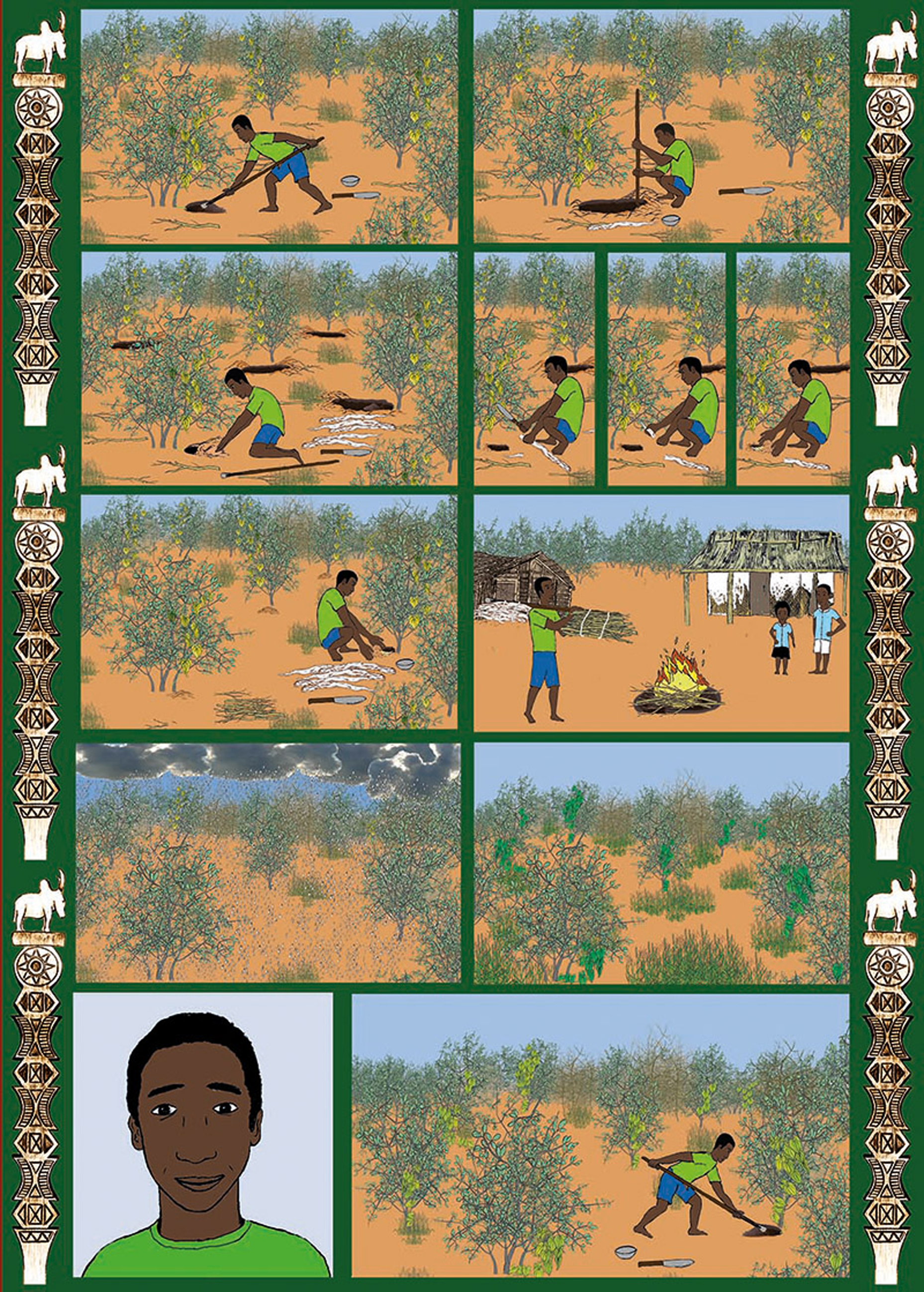

Supplement: S6 Fig — (TIF) [file pone.0217843.s006.tif]
