# Supplementary material for: The explanatory power of silent comics: An assessment in the context of knowledge transfer and agricultural extension to rural communities in southwestern Madagascar
Source: PLoS One. 2019 Jun 6;14(6):e0217843. doi: 10.1371/journal.pone.0217843 (PMC6553738; doi:10.1371/journal.pone.0217843)
Supplement: S1 Text — File consists of Tables A-D. (PDF) [file pone.0217843.s007.pdf]

S7 Text

Number of conducted interviews per comic within the six communities

Table A The number of conducted interviews per comic within the six communities during the second survey that aimed to evaluate locals' comprehension regarding the impact of unsustainable management practices on the environment and local livelihood. The comic on "samata" utilization was only distributed within communities of coastal villages as this spurge species mainly grows on the sandy soils of the coastal plain. The collection of wild yam, on the other hand, is far more common in the northern part of the Mahafaly Plateau wherefore comics on sustainable practices were distributed and evaluated only within three communities for which this topic is of utmost relevance.

**Community**	**Compost production**	**"samata" utilization**	**Wild yam harvest**	Total
Ampotaka	20		21	41
Andremba	21		22	43
Ankilibory	19	11		30
Efoetse	12	11		23
Marofijery	20	19		39
Miarintsoa	20		20	40
Total	112	41	63	216

Additional information on qualitative data analysis as well as posed questions and collected data types

The here conducted qualitative data analysis followed a content and framework analysis approach. In more detail, data on the comic acceptance by local communities assessed through group discussions during the first survey was evaluated qualitatively (Table A) by identifying general pattern of the respondents' answers and subjects for discussion that were noted by the moderator and the interviewers. Analysis were conducted through scanning primary data for words and phrases often used by the respondents to represent communities' general point of view as accurate as possible, but in a way that generalizing statements could be made.

Table B Posed questions at group discussions within the framework of the first survey to evaluate locals' comprehension on silent comics that show the impact of unsustainable practices and recommended sustainable practices on the environment and local livelihoods.

**Posed questions**	**Data type**
How did you find the comic?	Qualitative
Is there something you particularly liked on the comic or the drawing?	Qualitative
Is there anything you did not appreciate on the comic or the drawing?	Qualitative
Have you recognized something on the comic?	Qualitative
Have you noticed any differences between the two stories?	Binary (yes, no)
If yes, which one?	Qualitative

The majority of the data collected during the second survey included quantitative data. To facilitate data analysis, (response) codes were already defined in advance. This refers, for example, to the degree of respondents' comprehension of the illustrated practices, measured with a scale of 0 to 3 (categorical) by the interviewer or to the question if respondent had a prior encounter with research projects and/or NGOs (binary, yes/no). See Table B for detailed information. A further part of the data obtained during the second survey were

collected qualitatively, but could be quantified due to their strong uniformity and often repetitive responses as specific questions were asked which offered little scope for comprehensive and thematically diverse answers by the respondents. This primarily included a list of potential ecological or social consequences of continuing the current management practice or applying the recommended sustainable practice as well as challenges and opportunities for respondents of implementing the recommended practice. The quantification of these data was carried out by means of an abstraction and categorization of responses that followed the same meaning, but not the same wording. For instances, respondents' perception of consequences regarding recommended practice on compost production and its application to crops included statements such as better plant performance, better yield, better plant health, greener plants, etc.. All these statements followed the same meaning and were thus grouped into "Good plant performance". However, special care was taken to ensure that the reasoning of the statements was not lost during quantification. Therefore, depending on the topic of the comic and the question, statements were categorized in different fineness. Using the example of potential positive effects of a sustainable wild yam harvest, a distinction was made as to whether the respondent expected a possible increase in yield or a continuous yield, since this plays a decisive role when it comes to the sustainability aspect of a management practice.

Table C Collected data and posed questions at individual-based interviews during the second survey to evaluate locals' comprehension on silent comics that show the impact of unsustainable practices and recommended sustainable practices on the environment and local livelihoods.

**Collected data and posed questions**	**Data type**
Respondent name	Qualitative
Respondent sex (explanatory variable)	Binary, male/female
Respondent age (explanatory variable)	Integer, Minimum age = 11, maximum age = 60
Education level of respondent (explanatory variable)	Categorical,1-7 +++ 1 = no formal education, 2. = visited primary school, 3 = finished primary school, 4 = visited secondary school, 5 = finished secondary school, 6 = high school, 7 = University
Normal practice (explanatory variable)	Categorical, NP, UP, IP, RP +++ NP = no practice, UP = unsustainable practice, IP = improved practice, RP = recommended practice
Inference for extension (explanatory variable)	Binary, yes/no
Prior encounter with research project and/or NGO (explanatory variable)	Binary, yes/no
Understandability (response variable)	Categorical, 0-3 +++ 0 = not understood, 1 = partly understood, 2 = understood, 3 = completely understood
Number of stories recognized by respondent (response variable)	Categorical, LESS, EXACT, MORE
Understandability of story about unsustainable practice (response variable)	Categorical, 0-3 +++ 0 = not understood, 1 = partly understood, 2 = understood, 3 = completely understood
Understandability of story about recommended, sustainable practice (response variable)	Categorical, 0-3 +++ 0 = not understood, 1 = partly understood, 2 = understood, 3 = completely understood
Understandability of differences between both stories (response variable)	Categorical, 0-3 +++ 0 = not understood, 1 = partly understood, 2 = understood, 3 = completely understood
Willingness of adaption	Binary, yes/no
Perception of consequences of unsustainable practice (descriptive)	Qualitative
Perception of consequences of recommended, sustainable practice (descriptive)	Qualitative

The quantitative data on the socio-economic structure of the studied communities (e.g. age, gender or education level of respondents) were used as explanatory variables in our regression models. In addition, these parameters were used to calculate a structural evenness (SEve) and structural divergence (SDiv) of the studied communities by linking a respondent matrix with a respondent-socio-economic matrix considering gender, age, education level, INFEXT and PRENC as socio-economic individual parameters of a respondent (see material and method section, data analysis). In socio-economic and socio-demographic studies, factor analysis or principal component analysis (PCA) is often used to structure, simplify and visualize data sets by approximating a number of socio-economic variables such as gender, age, education or income, by a smaller number of meaningful linear combinations. Whereas applying PCA can lead to a reduction in the dimensionality of a data set, the here used approach to calculate an index on communities' structural divergence and evenness considers all parametric dimensions. However, to the best of our knowledge, no attempt has yet been made to quantify the structure of a group or community using an index. For this reason, we have used the field of community ecology, in which the use of formulas and indices to calculate structural diversity and structural divergence of plant and animal communities has been meanwhile a common practice. Thus, the statistical evaluation method applied on this data set represents a new approach to quantify socioeconomic structure of communities and its contribution to the openness and sensitivity of rural communities for a successful implementation of sustainable land management practices.

The third survey, which was conducted with the aim to evaluate to what extent the recommended practice was implemented by the interviewees over the past months, contained both quantitative (categorical and binary) and qualitative data (Table C). Since here recurring answers with similar meanings were given during the interviews, responses could be reduced and grouped to a few key words, wherefore these data could also be evaluated quantitatively.

Table D Collected data and posed questions at individual-based interviews during the third survey to evaluate locals' comprehension on silent comics that show the impact of unsustainable practices and recommended sustainable practices on the environment and local livelihoods.

**Collected data and posed questions**	**Data type**
Respondent name	Qualitative
Practiced between 2nd and 3rd survey	Binary, yes/no
If collected, which applied practice? (data subset)	Categorical, UP, IP, RP +++ UP = unsustainable practice, IP = improved practice, RP = recommended practice
Reason for applied practice	Qualitative
Challenges of applying RP?	Qualitative
Opportunities of applying RP?	Qualitative
Observation of people applying RP?	Binary, yes/no
Which person was observed?	Qualitative
Observation of people following UP?	Binary, yes/no
Which person was observed?	Qualitative
Which action was undertaken?	Qualitative
Knowledge sharing?	Binary, yes/no
If shared, to whom?	Qualitative
Reason for not sharing knowledge	Qualitative

The data collected on "Comic review through respondents and interviewers" that were presented in the form of a table (Table D) were based on a purely qualitative analysis and

rather followed a discourse analysis in which the naturally occurring talk and comments were compiled and ranked how often they were mentioned during group discussions and individual interviews. Overall, interviews took place in a very interactive way what made evaluation on the one hand complex, but also facilitated through a bi-directional knowledge transfer the process in finding general pattern in respondents' comprehension and awareness for their environment and natural resources on which their livelihood rely.
