# Supplementary material for: The explanatory power of silent comics: An assessment in the context of knowledge transfer and agricultural extension to rural communities in southwestern Madagascar
Source: PLoS One. 2019 Jun 6;14(6):e0217843. doi: 10.1371/journal.pone.0217843 (PMC6553738; doi:10.1371/journal.pone.0217843)
Supplement: S2 Text — (PDF) [file pone.0217843.s008.pdf]

Questionnaire Original langage (French)

I. Evaluation des Bandes dessinées sur l'utilisation durable des Samata, ignames sauvages et *l'utilisation de fumier dans les jardins familiaux* : discussion en groupe

A. Bande dessinée Samata

1. Comment avez-vous trouvé le BD sur le Samata?
2. Qu'est-ce que vous avez apprécié sur ce BD?
3. Y a-t-il des choses que vous n'avez pas appréciées sur ce BD?
4. Est-ce que vous avez reconnu quelque chose sur les dessins (objets, environnement,…)
5. Est-ce que vous avez remarqué des différences entre les deux histoires?<br>5.1. Si oui, quelles sont ces différences?

B. Bande dessinée Ignames sauvages

1. Comment avez-vous trouvé le BD sur le Baboky?
2. Qu'est-ce que vous avez apprécié sur cette BD?
3. Y a-t-il des choses que vous n'avez pas appréciées sur cette BD?
4. Est-ce que vous avez reconnu quelque chose sur les dessins (objet, environnement…)
5. Est-ce que vous avez remarqué des différences entre les deux histoires?<br>5.1. Si oui, quelles sont ces différences?

C. Bande dessinée Utilisation de fumier dans les jardins familiaux

1. Comment avez-vous trouvé le BD sur l'utilisation de fumier?
2. Qu'est-ce que vous avez apprécié sur cette BD?
3. Y a-t-il des choses que vous n'avez pas appréciées sur cette BD?
4. Est-ce que vous avez reconnu quelque chose sur les dessins (objet, environnement…)

5. Est-ce que vous avez remarqué des différences entre les deux histoires?<br>5.1. Si oui, quelles sont ces différences?

II. Evaluation des Bandes dessinées sur l'utilisation durable des Samata, ignames sauvages et *l'utilisation de fumier dans les jardins familiaux* : deuxième étape – entrevue individuelle

A. Bande dessinée Samata

1. Pouvez-vous me raconter l'histoire de la BD sur la collecte de Samata<br>(Non compris / Un peu compris/ Compris / Compris complétement)
2. Combien d'histoires trouvez-vous sur la BD ?
3. Pouvez-vous me raconter l'histoire sur la gauche<br>(non décris /Un peu décris / décris / décris complètement)
4. Pouvez-vous me raconter l'histoire sur la droite ?<br>(non décris /Un peu décris / décris / décris complètement)
5. Quelle est la différence entre les deux histoires?<br>(non décris /Un peu décris / décris / décris complètement)
6. D'habitude, comment collectez-vous les Samata?<br>Histoire sur la gauche/Histoire sur la droite/autres<br>Si autres idées? Lesquelles, Comment?
7. Quelle est la conséquence si les gens suivent l'histoire sur la gauche?
8. Quelle est la conséquence si les gens suivent l'histoire sur la droite?
9. Maintenant que vous êtes au courant de la différenc entre les deux histoires, comment allez-vous collecter les Samata?
10. Est que vous êtes au courant d'une parcelle d'essai sur la régénération de Samata mis en place par SuLaMa à Ambola?
11. Comment trouvez vois cette idée?<br>11.1 Pourquoi?
12. Avez d'autres idée comment cultiver les Samata?

B. Bande dessinée Ignames sauvages

1. Pouvez-vous me raconter l'histoire de la BD sur la collecte de Baboky ?<br>(Non compris / Un peu compris/ Compris / Compris complétement)
2. Combien d'histoires trouvez-vous sur la BD ?

3. Pouvez-vous me raconter l'histoire sur la gauche<br>(non décris /Un peu décris / décris / décris complètement)
4. Pouvez-vous me raconter l'histoire sur la droite ?<br>(non décris /Un peu décris / décris / décris complètement))
5. Quelle est la différence entre les deux histoires?<br>(non décris /Un peu décris / décris / décris complètement)
6. D'habitude, comment collectez-vous les Baboky?<br>Histoire sur la gauche/Histoire sur la droite/autres<br>Si autres idées? Lesquelles, Comment?
7. Quelle est la conséquence si les gens suivent l'histoire sur la gauche?
8. Quelle est la conséquence si les gens suivent l'histoire sur la droite?
9. Maintenant que vous êtes au courant de la difference entre les deux histoires, comment allez-vous collecter les Baboky?
10. Est que vous êtes au courant de la culture/régénération de Baboky/Ovy dans la forêt d'Ampotaka et aux champs d'Andremba
11. Comment trouvez vois cette idée?<br>12. Pourquoi?
13. Avez d'autres idée comment cultiver les Samata le Baboky ou le Ovy?

C. Bande dessinée Utilisation de fumier dans les jardins familiaux

1. Pouvez-vous me raconter l'histoire de la BD sur l'utilisation de fumier<br>(Non compris / Un peu compris/ Compris / Compris complétement
2. Combien d'histoires trouvez-vous sur la BD ?
3. Pouvez-vous me raconter l'histoire sur la gauche<br>(non décris /Un peu décris / décris / décris complètement)
4. Pouvez-vous me raconter l'histoire sur la droite ?<br>(non décris /Un peu décris / décris / décris complètement)
5. Quelle est la différence entre les deux histoires?<br>(non décris /Un peu décris / décris / décris complètement)
6. D'habitude, comment cultivez vous les légumes dans vos jardins?<br>Histoire sur la gauche/Histoire sur la droite/autres<br>a. Si autres idées? Lesquelles, Comment?
7. Quelle est la conséquence si les gens suivent l'histoire sur la gauche
8. Quelle est la conséquence si les gens suivent l'histoire sur la droite?
9. Maintenant que vous êtes au courant de la différence entre les deux histoires,

comment allez-vous cultiver les légumes dans vos Jardins?
10. Etes-vous au courant des essai sur la préparation de composte réalisé par SuLaMa?
11. Comment trouvez vois cette idée?<br>11.1 Pourquoi
12. Avez d'autres idée comment préparer le Composte?

III. Evaluation des Bandes dessinées sur l'utilisation durable des Samata, ignames sauvages et *l'utilisation de fumier dans les jardins familiaux* : troisième étape – entrevue individuelle-Suivi

A. Bande dessinée Samata

1. Avez vous collecté des Samata depuis la dernière fois qu'on vous a interviewé?<br>1.1. (Si oui) quelle méthode avez-vous utilisé (histoire de gauche / (histoire de gauche / autres)<br>- Est-ce que c'est nouveau pour vous ?<br>- Si oui, pourquoi avez-vous changé votre méthode?<br>- Comment avez-vous trouvez la mise en pratique de cette méthode?<br>1.2. (Si non)) pourquoi?
2. Avez-vous déjà vu quelqu'un d'autres utiliser la collecte durable des Samata ? (Si oui) (Voisins / membre de la famille / amis / autres = …………………………….)<br>Si oui, qu'avez-vous fait ?
3. Qu'allez-vous faire si vous voyez quelqu'un appliquant la méthode de collecte sur la gauche?
4. Qu'allez-vous faire si vous voyez quelqu'un appliquant la méthode de collecte sue la droite?
5. Si vous suivez la méthode de collecte sur la droite, quelle est votre attente?

B. Bande dessinée ignames sauvages

1. Avez vous collecté des Baboky depuis la dernière fois qu'on vous a interviewé?<br>1.1. (Si oui) quelle méthode avez-vous utilisé (histoire de gauche / (histoire de gauche / autres)<br>- Est-ce que c'est nouveau pour vous ?<br>- Si oui, pourquoi avez-vous changé votre méthode?<br>- Comment avez-vous trouvez la mise en pratique de cette méthode?<br>1.2. (Si non)) pourquoi?
2. Avez-vous déjà vu quelqu'un d'autres utiliser la collecte durable des Baboky/Ovy ?

(Si oui) (Voisins / membre de la famille / amis / autres = …………………………….)<br>Si oui, qu'avez-vous fait ?
3. Qu'allez-vous faire si vous voyez quelqu'un appliquant la méthode de collecte sur la gauche?
4. Qu'allez-vous faire si vous voyez quelqu'un appliquant la méthode de collecte sur la droite?
5. Si vous suivez la méthode de collecte sur la droite, quelle est votre attente?

C. Bande dessinée Compost comics

1. Avez-vous cultivé des légumes dans vos jardins depuis la dernière fois qu'on vous a interviewé?<br>1.1. (Si oui) quelle méthode avez-vous utilisé (histoire de gauche / (histoire de gauche / autres)<br>- Est-ce que c'est nouveau pour vous ?<br>- Si oui, pourquoi avez-vous changé votre méthode?<br>- Comment avez-vous trouvez la mise en pratique de cette méthode?<br>1.2. (Si non)) pourquoi?
2. Avez-vous déjà vu quelqu'un d'autres cultiver des légumes avec du Composte?<br>(Si oui) (Voisins / membre de la famille / amis / autres = …………………………….)<br>Si oui, qu'avez-vous fait ?
3. Qu'allez-vous faire si vous voyez quelqu'un cultiver des légumes avec la méthode sur la gauche?
4. Qu'allez-vous faire si vous voyez quelqu'un appliquant la méthode de collecte sur la droite ?
5. Si vous suivez la méthode de collecte sur la droite, quelle est votre attente

Questionnaire translated in English

I. Evaluation of comics on the sustainable use of Samata (*Euphorbia stenoclada*) and wild yams (Dioscorea spp.): group discussion

A. Samata comis

1. How did you find the Samata comics?
2. What did you like about the comics?
3. Is there anything you did not like on the comics?

4. What have you recognized on the comics?
5. Have you seen any difference between the two stories?<br>5.1. If the majority say of the participants says YES, (What is the difference?)

B. Yam comics

1. How did you find the Baboky comics?
2. What did you like about the comics?
3. Is there anything you did not like on the comics?
4. What have you recognized on the comics?
5. Have you seen any difference between the two stories?<br>5.1. If the majority say of the participants says YES, (What is the difference?)

C. Compost comics

1. How did you find the comics about the use of manure?
2. What did you like about the comics?
3. Is there anything you did not like on the comics?
4. What have you recognized on the comics?
5. Have you seen any difference between the two stories?<br>5.1. If the majority say of the participants says YES, (What is the difference?)

II. Evaluation of comics on the sustainable use of Samata (*Euphorbia stenoclada*) and wild yams (Dioscorea spp.): second step - individual interview

A. Samata comics

1. Can you tell me the stories about Samata comics you received last time?<br>(Did not understand / Partially understood / understood / Completely understood)
2. Are you aware that there are two stories on the comics?
3. Can you tell me the story on the left side?<br>(not described at all /described partially / described / completely described)
4. Can you tell me the story on the right side?<br>(Could not describe /described partially / described / completely described)
5. What is the then the difference between the two stories?<br>(not described at all /described partially / described / completely described)

6. How do you usually do when harvesting Samata?<br>(left story / right story / other ideas)<br>6.1. (If other ideas other ideas), which ones/How?
7. What is the consequence of people follow the left story on Samata harvesting?
8. What is the consequence of people follow the left story on Samata harvesting?
9. Now that you know the difference between the stories, how would you harvest Samata in the future?
10. Are you aware of the trial on Samata regeneration conducted in Ambola by SuLaMa?
11. How do you find this idea?<br>11.1. Why?
12. Do you have other idea how to grow Samata?

B. *Yam comics*

1. Can you tell me the stories about Baboky comics you received last time? (Did not understand / Partially understood / understood / Completely understood)
2. Are you aware that there are two stories on the comics?
3. Can you tell me the story on the left side?<br>(not described at all /described partially / described / completely described)
4. Can you tell me the story on the right side?<br>(Could not describe /described partially / described / completely described)
5. What is the then the difference between the two stories?<br>(not described at all /described partially / described / completely described)
6. How do you usually do when harvesting Baboky?<br>(left story / right story / other ideas)<br>(If other ideas other ideas), which ones/How?
7. What is the consequence of people follow the left story on Samata harvesting?
8. What is the consequence of people follow the left story on Samata harvesting?
9. Now that you know the difference between the stories, how would you harvest Samata in the future?
10. Are you aware of the trial any cultivation of Baboky in the forest (Ampotaka) or Ovy on field (Andremba)?
11. How do you find this idea?<br>Why?
12. Do you have other idea how to grow Baboky or Ovy?

C. *Compost comics:*

1. Can you tell me the stories about the homegarden comics you received last time?<br>(Did not understand / Partially understood / understood / Completely understood)
2. Are you aware that there are two stories on the comics?
3. Can you tell me the story on the left side?<br>(not described at all /described partially / described / completely described)
4. Can you tell me the story on the right side?<br>(Could not describe /described partially / described / completely described)
5. What is the then the difference between the two stories?<br>(not described at all /described partially / described / completely described)
6. How do you usually do when growing vegetable in your homegarden?<br>(left story / right story / other ideas)<br>(If other ideas other ideas), which ones/How?
7. What is the consequence of people follow the left story on baboky harvesting?
8. What is the consequence of people follow the right story on baboky harvesting?
9. Now that you know the difference between the stories, how would you grow vegetable in your homegarden in the future?
10. Are you aware about any trial on how to prepare compost?
11. How do you find this idea?<br>Why?
12. Do you have other idea how to prepare compost?<br>If yes, how?

III. Evaluation of comics on the sustainable use of Samata (*Euphorbia stenoclada*) and wild yams (Dioscorea spp.): third step - individual interview: third step (follow-up individual interview)

A. Samata comics

1. Have you Harvested Samata since the last time we met?<br>1.1 (If yes) which harvesting practice have you used (left story / right story / other methods)<br>- Is this new to you?<br>- If yes, why have you changed your old practice?<br>- How did you find the practice of this method?<br>1.2. (If no harvest of Samata since the last time) why?
2. Have you seen anybody else applying the Sustainable harvest of Samata?<br>(if yes) (Neighbors / family members / friends / other = …………………………….……….)<br>2.1. Have you seen anybody else applying the unsustainable (left comic) harvest of Samata?<br>If yes, what have you done?
3. What do you do if you see anybody else following the harvesting practice on the left story?
4. What do you do if you see anybody else following the harvesting practice neither left nor right story?
5. If you practiced the harvesting practice of Samata on the right story, what is your expectation?

B. Yam comics

1. Have you harvested Yams (Baboky/ovy) since the last time we met?<br>1.1. (If yes) which harvesting practice have you used (left story / right story / other methods)<br>- Is this new to you?<br>- If yes, why have you changed your old practice?<br>- How did you find the practice of this method?<br>1.2. (If no harvest of yam since the last time) why?
2. Have you seen anybody else using the re-planting Baboky in the forest?<br>(if yes) (Neighbors / family members / friends / other = …………………………….……….)<br>3. Have you seen anybody else applying the unsustainable (left comic) harvest of Samata?<br>If yes, what have you done?
4. What do you do if you see anybody else following the harvesting practice on the left story?
5. If you practiced the harvesting practice of Baboky on the right story, what is your expectation?

C.Compost comics

1. Have you already planted vegetable in your garden since the last time we met?<br>1.1. (If yes) which method (left story / right story / other methods)<br>- Is this new to you?<br>- If yes, why have you changed your old practice?<br>- How did you find the practice of this method?<br>1.2. (If no) why not?
2. Have you seen anybody else applying the new methods (the right story)?<br>(if yes) (Neighbors / family members / friends / other = ……………………….…………)<br>3. Have you seen anybody else still applying the old methods (left comic)?<br>If yes, what have you done?
4. What do you do if you see anybody else still following the left story?
5. If you practiced to cultivate vegetables in your garden like the right story, what is your expectation?
