# Supplementary material for: The explanatory power of silent comics: An assessment in the context of knowledge transfer and agricultural extension to rural communities in southwestern Madagascar
Source: PLoS One. 2019 Jun 6;14(6):e0217843. doi: 10.1371/journal.pone.0217843 (PMC6553738; doi:10.1371/journal.pone.0217843)
Supplement: S3 Text — File consists of Table E. (PDF) [file pone.0217843.s009.pdf]

S9 Text

Table E Factors influencing positively (+) or negatively (-) the comprehension and awareness of respondents in six communities of the Mahafaly region in southwestern Madagascar.

	Compost production	"samata" utilization	Wild yams harvest
**Comprehension**	*General understandability*		
	**Normal practice** (1.00, UP +, RP +)**		Inference for extension (0.23, +)
	**Education level** (0.46, +)*		Normal practice (0.23, UP -, RP +)
	Inference for extension (0.27, -)		
	Prior encounter (0.13, -)		
	Number of stories illustrated in the comic (two stories intended)		
	**Gender** (1.00, male -)**	Prior encounter (0.21, +)	Normal practice (0.65, UP +, RP -)
	**Age x Gender** (1.00, male +)*	Age (0.18, +)	Prior encounter (0.58, +)
	Location (0.49, Plateau, -)	Normal practice (0.17, RP +)	Education level (0.13, -)
	Normal practice (0.23, UP -, RP -)		
	Inference for extension (0.11, +)		
	Recognition of differences between unsustainable and recommended practice		
	**Normal practice** (1.00, UP +, RP +)***	Normal practice (0.69, RP -)	Normal practice (0.25, UP -, RP +)
	**Education level** (1.00, +)**		Inference for extension (0.23, +)
**Awareness**	*Perception about unsustainable practice illustrated in comic*		
	**Normal practice** (1.00, UP +, RP +)***	Normal practice (0.24, RP +)	Inference for extension (0.29, +)
		Location (0.22, Plateau -)	
	Perception about recommended practice illustrated in comic		
	**Education level** (1.00, +)***	Normal practice (0.29, RP -)	Inference for extension (0.29, +)
	Normal practice (1.00, UP +, RP +)**		

Filtering of factors was done by model averaging and usage of relative variable importance (in bracket). Statistically significant influencing factors using $p \leq 0.05$ as threshold for significance are presented in bold. Stars indicate statistical significance of estimated mean generated through model-averaging using the threshold of $p \leq 0.001$ (***), $p \leq 0.01$ (**), $p \leq 0.05$ (*). Effects of normal practice of Malagasy farmers included unsustainable (UP) or recommended practice (RP).
